# Distinct roles for motor cortical and thalamic inputs to striatum during motor skill learning and execution

**DOI:** 10.1126/sciadv.abk0231

**Published:** 2022-02-25

**Authors:** Steffen B. E. Wolff, Raymond Ko, Bence P. Ölveczky

**Affiliations:** Department of Organismic and Evolutionary Biology and Center for Brain Science, Harvard University, Cambridge, MA 02138, USA.

## Abstract

The acquisition and execution of motor skills are mediated by a distributed motor network, spanning cortical and subcortical brain areas. The sensorimotor striatum is an important cog in this network, yet the roles of its two main inputs, from motor cortex and thalamus, remain largely unknown. To address this, we silenced the inputs in rats trained on a task that results in highly stereotyped and idiosyncratic movement patterns. While striatal-projecting motor cortex neurons were critical for learning these skills, silencing this pathway after learning had no effect on performance. In contrast, silencing striatal-projecting thalamus neurons disrupted the execution of the learned skills, causing rats to revert to species-typical pressing behaviors and preventing them from relearning the task. These results show distinct roles for motor cortex and thalamus in the learning and execution of motor skills and suggest that their interaction in the striatum underlies experience-dependent changes in subcortical motor circuits.

## Teaser

Motor cortex guides plasticity in thalamostriatal circuits during skill learning.

## INTRODUCTION

**Fig. 1.**
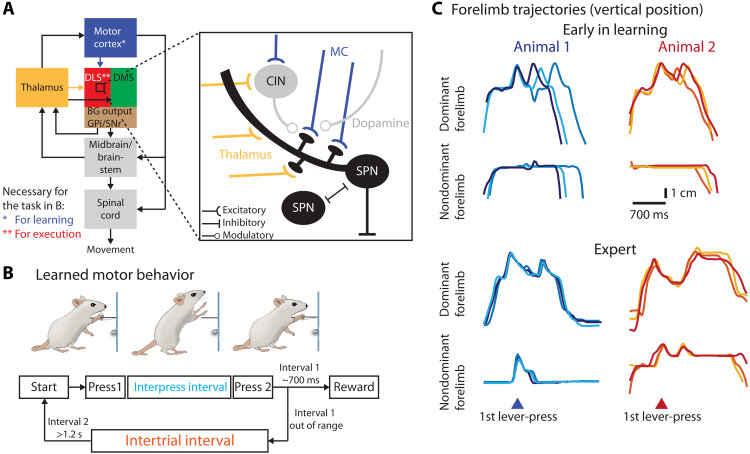
Probing the role of excitatory inputs to striatum in motor skill learning and execution. (**A**) Schematic of the probed motor network with the BG subdivided into DLS and dorsomedial (DMS) striatum and the output nuclei globus pallidus internus (GPi) and substantia nigra pars reticulata (SNr). Somatosensory cortical projections to DLS, motor cortical projections to other BG nuclei, cerebellar inputs to thalamus, and dopaminergic midbrain projections to DLS/DMS are omitted for clarity. Expanded view: The main excitatory inputs to DLS from motor cortex (MC) (blue) and thalamus (yellow) target overlapping populations of SPNs, cholinergic (CIN), and other interneurons (omitted for clarity). (**B**) Behavioral paradigm to test the role of the DLS and its excitatory inputs during learning and execution of complex movement patterns. Rats are rewarded for pressing a lever twice with a specific delay [interpress interval (IPI)]. After unsuccessful trials, animals must refrain from pressing the lever for 1.2 s [intertrial interval (ITI)] to initiate a new trial. (**C**) Forelimb trajectories of two example animals early (top) and late in training (bottom). Shown are vertical positions of dominant (executing the first lever-press) and nondominant forelimbs for three trials each (randomly selected rewarded trials after unrewarded trials).

Whether tying our shoelaces or hitting a volleyball serve, we rely on our brain’s ability to learn and reliably generate stereotyped task-specific movement patterns. These processes depend on the interplay between various cortical and subcortical brain areas (Fig. 1A) (*1*, *2*). Yet, the roles of the specific circuits and how they interact during the acquisition and execution of such motor skills are not well understood (*1*, *2*). The striatum, the main input nucleus of the basal ganglia (BG), is an important nexus in the distributed mammalian motor network and one through which cortical and subcortical circuits interact (*3*–*5*). Its sensorimotor arm [dorsolateral striatum (DLS) in rodents] receives most of its excitatory input from sensorimotor cortex and thalamus (*3*–*5*). Striatal spiny projection neurons (SPNs) in DLS can influence movements through their actions on BG output nuclei in two principal ways: by modulating motor cortical activity via the BG-thalamo-cortical pathway (*4*–*6*) and by influencing brainstem and midbrain motor circuits via direct projections from BG output nuclei (Fig. 1A) (*4*, *7*).

While the function of the DLS has been extensively probed in various forms of learning, including action-outcome and stimulus-response learning (*8*, *9*), its contributions to the acquisition and generation of the complex movement patterns that underlie many motor skills have received far less attention. Much of the focus has instead been on tasks that can be mastered by executing (and/or slightly adapting) movements or actions readily expressed by the animals at the beginning of learning, such as ballistic forelimb swipes directed at a lever (*10*–*12*) or locomotion (*13*). While their detailed kinematics are rarely measured and analyzed, we note that these actions can be very similar across members of a species, and the DLS is not necessary for generating their detailed time-varying structure, even when they are part of an unnatural experimenter-defined task (*10*, *12*–*15*). As in our previous paper (*15*), we here refer to these actions as “species-typical,” inspired by a term from ethology (*16*), setting them apart from idiosyncratic task-specific movement patterns acquired over extended periods of training in complex motor tasks.

To study the role of the DLS in the acquisition and execution of novel and task-specific movement patterns, we used a paradigm that trains rats to produce idiosyncratic and highly stereotyped behaviors with complex learned kinematic structure (Fig. 1, B and C, and movie S1) (*17*). Using this task (Fig. 1, B and C), we recently showed that the DLS is essential for generating the learned movement patterns, with DLS lesions causing animals to revert to species-typical lever-press movements used early in learning (*15*).

Comparing our result to other learned behaviors where the detailed movement patterns are largely unaffected by DLS lesions (*10*, *12*–*14*) suggests that its role depends on the specific challenges associated with mastery of a task. While DLS is essential for generating the fine-grained and time-varying kinematic structure of learned task-specific movement patterns (*15*), it is not needed for expressing species-typical behaviors (*10*, *13*, *14*), a distinction nicely encapsulated by the comparison of our task to a recent study by Jurado-Parras *et al.* (*13*), in which locomotion and waiting are adapted to solve a treadmill-based task. While DLS lesions can affect the speed of locomotion in this paradigm (*13*), consistent with a role for the DLS in modifying the vigor of such behaviors (*13*, *14*, *18*), the locomotor behavior is preserved, a qualitatively different result from the complete loss of task-specific learned kinematic structure following DLS lesions in our task (*15*). However, while prior results (*15*, *19*) have demonstrated a critical role for the DLS in specifying the fine-grained details of task-specific learned movement patterns, little is known about whether and how inputs from motor cortex and thalamus support this specific function and how these inputs interact with the DLS during learning to drive plasticity in subcortical motor circuits [although see (*20*–*23*)].

What we do know is that motor cortex serves as a major source of input to the DLS and plays a critical role in many forms of motor learning (*2*, *3*, *5*, *24*). The idea that the formation and storage of motor memories rely on experience-dependent plasticity at corticostriatal synapses has received some experimental support (*21*, *22*). The generality of this notion, however, has been challenged by our recent studies, showing that the DLS-dependent learned motor skills we train can survive motor cortex lesions (*15*, *17*).

The very same learned movement patterns that survive lesions of motor cortex cannot be acquired without it (*17*), suggesting a role for motor cortex in learning that is independent of its role in control (*25*, *26*). This raises the possibility that motor cortical inputs to DLS, rather than storing crucial aspects of the acquired behavior, guide learning-related plasticity within the striatum (*27*). Alternatively, motor cortex could enable learning by informing plasticity in its other projection targets, such as the midbrain, brainstem, and/or spinal circuits (*26*, *28*, *29*) or other parts of the BG such as the subthalamic nucleus (*30*) or the globus pallidus externa (*31*).

If critical learning–related circuit plasticity occurs within the DLS, but not at motor cortical inputs, then it may manifest as plasticity either between SPNs and/or at other inputs to the DLS. The main inputs besides that from motor cortex are from somatosensory cortex and thalamus (Fig. 1A) (*3*, *32*, *33*). While there is some evidence for a role of somatosensory cortex in motor learning (*34*, *35*), little is known about the function of thalamic inputs to the striatum, despite their being comparable in strength and numbers to those from cortex (*33*). While a number of thalamic nuclei send collaterals to the striatum (*32*), including motor (*36*, *37*) and somatosensory nuclei (*38*, *39*), the parafascicular nucleus (Pf) and the rostral intralaminar nuclei (rILN) (*32*, *33*) are the main sources of thalamic input to the striatum. These regions of the thalamus relay information predominantly from subcortical circuits (*32*, *33*, *40*) and notably the cerebellum (*41*). The thalamostriatal projections from the Pf and rILN have been implicated in modulating the activity of cortical inputs to striatum via cholinergic interneurons (Fig. 1A), in the regulation of attention and behavioral flexibility, and in providing information to the BG about the behavioral state and context for rapid behavioral adaptations (*23*, *42*, *43*). A study recently implicated certain thalamostriatal projections [Pf to dorsomedial striatum (DMS) and ventral posterior nucleus (VP) to DLS] in the initiation and execution of learned action sequences (*38*), yet the role of the inputs from the Pf and rILN to the DLS in motor skill learning and execution has not been parsed.

To address the question of how DLS and its various inputs underlie the acquisition and execution of learned motor skills, we turned to the paradigm mentioned above, which trains highly stereotyped, idiosyncratic, and task-specific movement patterns in rats (Fig. 1, B and C, and movie S1) (*17*). Using a variety of targeted circuit manipulations, we show that the DLS and the motor cortical neurons that project to it are required for learning the behaviors we train, while somatosensory cortex is not. We further show that synaptic changes in the DLS are an essential part of the underlying motor memory and that thalamic neurons projecting to the DLS are necessary for learning and executing the task-specific movement patterns. These results provide important new insights into the interplay between motor cortex, thalamus, and striatum, and how it underlies the acquisition and production of task-specific movement patterns.

## RESULTS

### The acquisition of complex movement patterns requires the DLS but not the DMS

To probe the role of the striatum and its inputs in the acquisition and control of task-specific movement patterns, we used our paradigm mentioned above, in which rats are rewarded with water for pressing a lever twice within a specific time interval [interpress interval (IPI); target: 700 ms] (Fig. 1B and movie S1) (*17*). Over the course of a month-long operant shaping procedure, rats develop de novo idiosyncratic movement patterns, which include various motor elements in addition to the lever-presses themselves, allowing them to keep the prescribed time between the presses (Fig. 1C and movie S1). These emerging movement patterns become increasingly complex, fluid, spatiotemporally precise, and stereotyped [Fig. 1C and movie S1; for a detailed characterization of the task and the development of the learned movement patterns, see (*17*)].

**Fig. 2.**
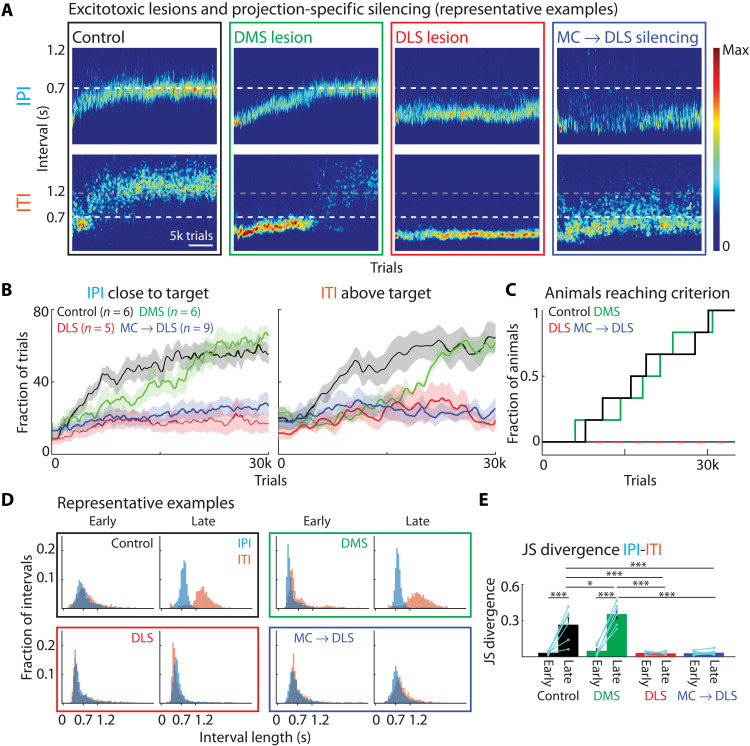
DLS and DLS-projecting motor cortex neurons are required for motor skill learning. (**A**) Effects of pre-training manipulations on task performance throughout learning for four conditions: DLS control injection, DMS excitotoxic lesions, DLS excitotoxic lesions, and silencing of DLS-projecting motor cortex neurons (MC → DLS) (see Results, Materials and Methods, and fig. S1B for details). Shown are heatmaps of IPI and ITI probability distributions for representative animals. (**B**) Population performance for manipulations as in (A). Controls include animals with DLS control injections and GFP expression in DLS-projecting motor cortex neurons (*n* = 3 each; fig. S1, F and G). Left: Fraction of trials with IPIs close to the target (700 ms ± 20%). Initially, DMS-lesioned animals have lower learning rates than controls [repeated measures analysis of variance (RM-ANOVA) on blocks of 3000 trials: blocks 3000 to 6000 (*P* = 0.037), 6000 to 9000 (*P* = 0.013)] but show no differences or higher performance late in training. For detailed statistical comparison between all groups, see table S1. Right: Fraction of trials with ITIs above threshold (1.2 s) (fig. S1C and table S1). (**C**) Fraction of animals reaching the learning criterion and number of trials needed (Materials and Methods) (*17*). Comparing control and DMS animals (DLS and MC → DLS animals did not reach criterion) shows no significant difference in number of trials (unpaired *t* test; *P* = 0.922) or the cumulative curves (KS test; *P* = 1). (**D**) Distributions of durations between lever-presses for animals shown in (A) early (first 2000 trials) and late (trials 30,000 to 32,000) in training. (**E**) Dissimilarity between IPI and ITI distributions early and late in training as measured by the Jensen-Shannon (JS) divergence. For statistical details, see table S2 and fig. S3A for further comparison of the manipulations. Bars show means, dots show individual animals, and error bars show SEM. *P < 0.05 and ***P < 0.001.

To directly probe the contributions of the DLS to learning these task-specific movement patterns, we performed excitotoxic lesions in naïve animals and compared them to lesions of the DMS (Fig. 2A and fig. S1A), which has been implicated in the early phases of learning in other motor tasks (*9*, *11*, *22*, *44*). Rats were trained in our automated training system as described above (Fig. 1B) (*17*). We compared the performance of lesioned animals to a cohort receiving DLS-targeted control injections [retrobeads or retrograde Cre-expressing adeno-associated viral vectors (AAVs), combined with Cre-dependent AAVs in motor cortex for green fluorescent protein (GFP) expression in DLS-projecting motor cortex neurons (see Materials and Methods); the controls were combined because they showed no difference in learning (*n* = 3 each; for the learning curves for the individual control cohorts, see fig. S1 (F and G)]. Control animals learned, over weeks of training, to produce IPIs around the prescribed 700-ms target (Fig. 2, A and B; fig. S1, C and D; and fig. S2A), developing idiosyncratic stereotyped movement patterns as previously described (Figs. 1C and 2, A to C, and fig. S1, C and D) (*15*, *17*). All control animals reached our prespecified performance criteria (*17*) for mastering the task, in less than 30,000 trials [mean IPI within ±10% of target (700 ms) and coefficient of variation (CV) of the IPI distribution of less than 0.25; Fig. 2, A to C, and fig. S1, C and D]. In line with this, control animals also mastered an additional aspect of the task structure: They learned to withhold lever-pressing for 1.2 s [intertrial interval (ITI)] after unsuccessful trials before initiating a new trial (Materials and Methods; Figs. 1B and 2, A, B, D, and E; and figs. S1, C and D, and S2A) (*17*). DMS-lesioned animals all reached our learning criteria after a similar number of training trials as control animals {19,085 ± 8519 (DMS) versus 18,620 ± 8914 (control) trials (means ± SD); no significant difference [unpaired *t* test (*P* = 0.922) and Kolmogorov-Smirnov (KS) test on cumulative curves (*P* = 1), details in the caption of Fig. 2C]; Fig. 2, A to C, and figs. S1, C and D, and S2B}. The performance of DMS-lesioned animals, however, was initially significantly lower than those of control animals (Fig. 2B, fig. S1C, and table S1). This is consistent with studies on action-outcome and rotarod learning, which have demonstrated a deficit in the early phases of learning after DMS lesions (*44*). This initial deficit did not prevent the animals from learning our task: All DMS-lesioned animals caught up with the control animals and reached our prespecified learning criteria (*17*) after similar amounts of training (Fig. 2, B and C), eventually showing comparable performance across all our measures (Fig. 2, B and E; figs. S1, C and D, and S2E; and table S1).

In stark contrast, none of the DLS-lesioned animals reached criterion performance in our task after 30,000 trials (Fig. 2, A to C, and fig. S2C). A subset of animals for which training was extended for more than 60,000 trials also failed to reach our criteria (fig. S1E). Furthermore, while both control and DMS-lesioned animals learned the distinction between the IPI and the ITI (Fig. 2, D and E, and fig. S3A), indicating that they learned the task structure in addition to acquiring de novo movement patterns, DLS-lesioned animals did not distinguish these intervals as indicated by overlapping IPI and ITI distributions (Fig. 2, D and E, and fig. S3A). Together, our results show that the DLS is necessary for learning the task-specific movement patterns and the underlying task structure. DMS, on the other hand, can contribute to early phases of learning, but is not strictly required for acquiring or mastering any aspect of the task.

### Learning requires DLS-projecting motor cortex neurons

Our finding that DLS-lesioned animals have learning deficits (Fig. 2) similar to animals with motor cortex lesions (*17*) suggests the possibility that motor cortex informs learning-related plasticity in subcortical control circuits through its projections to the DLS. Alternatively, motor cortex could also exert its “tutoring” through its projections to control circuits in the midbrain, brainstem, and spinal cord and/or other BG nuclei, such as the subthalamic nucleus or the globus pallidus externa (Fig. 1A) (*7*, *26*, *29*–*31*). To address the role of motor corticostriatal projections in learning, we used an intersectional viral approach (Fig. 2A and fig. S1B) to silence DLS-projecting motor cortex neurons in naïve (untrained) animals. We injected retrogradely transported viruses expressing Cre-recombinase—either canine adenovirus type 2 (CAV-2) (*45*) or retrogradely transported AAV (rAAV) (*46*)—into the DLS. In addition, we injected a locally infecting AAV, conditionally coexpressing Tetanus Toxin Light Chain (TeLC) (*47*) and GFP in a Cre-dependent manner into motor cortex. TeLC cleaves the synaptic protein VAMP2, preventing membrane fusion of synaptic vesicles and release of neurotransmitters, effectively silencing neuronal output (*47*). Our two-component viral approach restricts TeLC expression to motor cortex neurons with axon terminals in the DLS. Note, however, that these neurons also send collaterals to other brain areas [e.g., different cortices, multiple (motor) thalamic nuclei, the spinal cord, or the pons] (*29*, *48*, *49*), which might contribute to their function in our task. Despite this caveat, this projection-specific silencing approach is far more selective than broad lesions of motor cortex (*17*). Not only is the local circuitry preserved but populations of callosal (*50*), cortico-subthalamic (*51*), and especially a large fraction of corticothalamic (*29*, *52*) neurons are also spared. We estimate our silencing efficiency to be at about 50% of DLS-projecting motor cortex neurons in the infected areas (compared to co-injected retrobeads; see Materials and Methods; fig. S1B). We used this approach to silence neurons in motor cortex that project to the DLS in a cohort of naïve animals (MC → DLS). Similar to DLS-lesioned (Fig. 2, A to C, and fig. S1, C and D) and motor cortex–lesioned animals (*17*), MC → DLS silencing animals failed to learn the task (Fig. 2, A to C, and figs. S1, C and D, S2D, and S3E). They did not reach the learning criteria for the IPI within 30,000 trials (Fig. 2C and fig. S1E), with a subset of animals undergoing extended training and failing to reach the criteria even after 60,000 trials (fig. S1E). While MC → DLS animals, on average, reach slightly higher IPIs than DLS-lesioned animals, both groups show similar deficits in all other measures (Fig. 2, B and E; figs. S1, C and D, and S3E; and table S1). MC → DLS animals also failed to learn the task structure, reflected by the lack of a distinction between IPI and ITI intervals (Fig. 2, D and E, and fig. S3A). To verify that these effects were due to silencing DLS-projecting motor cortex neurons rather than to unspecific Cre-independent TeLC expression, we injected another cohort of animals with the Cre-dependent TeLC AAV into motor cortex (with no retrograde virus in DLS; fig. S1, F and G). These animals showed no learning deficits (figs. S1, F and G). Together, these results show that DLS-projecting motor cortex neurons are necessary for learning the task-specific movement patterns we train, consistent with motor cortex guiding or, otherwise, enabling plasticity in subcortical motor circuits through direct projections to the DLS.

Notably, motor cortex is not the only source of cortical projections to the DLS, with somatosensory cortex providing the other main input (*3*). While there is evidence for a role of somatosensory cortex in some forms of motor learning (*34*, *35*), it remains an open question whether it exerts its influence mainly via its motor cortical (*35*), striatal (*53*), or spinal projections (*54*) and whether it provides contextual information and/or sensory feedback important for our task (*55*). To directly test a potential role in the learning process, we lesioned somatosensory cortex in a separate cohort of naïve animals (fig. S4). In contrast to animals with motor cortex lesions (*17*) or silenced motor cortex neurons projecting to DLS (Fig. 2), these animals did not show any impairment in learning (figs. S4 and S3, B and F). All animals with somatosensory cortex lesions learned our task and reached our learning criteria after similar amounts of training as control animals [14,434 ± 8439 (somatosensory cortex) versus 18,620 ± 8914 (control) trials (means ± SD); unpaired *t* test (*P* = 0.438) and KS test on cumulative curves (*P* = 0.549); fig. S4G]. This result shows that somatosensory cortex and its inputs to the DLS, in contrast to DLS-projecting motor cortex neurons, are not necessary for learning the motor skills we train.

### Reversal of long-term synaptic plasticity in DLS disrupts performance

Once animals learn the stereotyped movement patterns required for our task, they are expressed in largely unaltered form over long periods of time (*17*), indicating the formation of stable motor memories. Prior studies have suggested motor skill learning–related plasticity in motor cortex (*24*, *56*, *57*) and/or at synapses between the motor cortex and striatum (*21*, *22*). However, while this plasticity might play a role during the initial learning process, neither of these neural substrates is required for executing the behaviors we train (*17*). The crucial involvement of the DLS in both motor sequence learning (Fig. 2) and execution (*15*) suggests that the memory of the learned skills may be stored, in part at least, in the DLS, but at synapses distinct from those formed by motor cortical inputs.

**Fig. 3.**
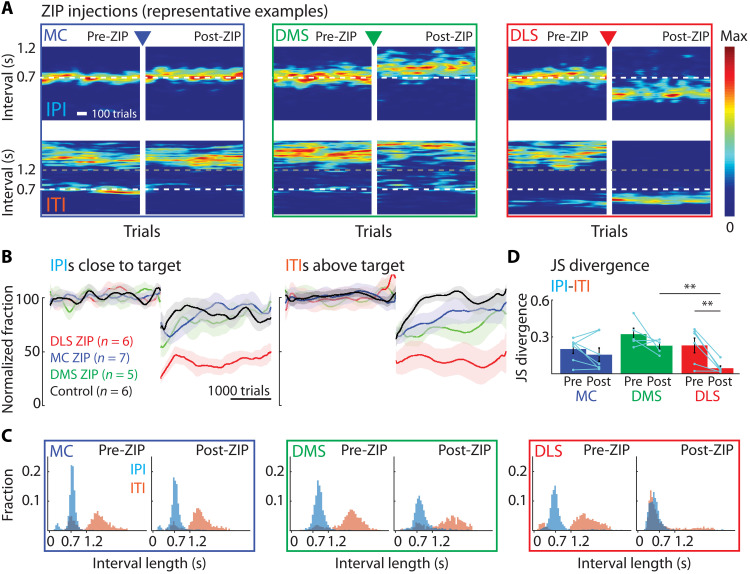
Pharmacological reversal of plasticity at excitatory synapses in DLS, but not DMS or motor cortex, disrupts execution of the learned skill. (**A**) Representative expert animals injected with ZIP, an inhibitor of an enzyme necessary for maintaining synaptic plasticity at excitatory synapses, in either motor cortex, DMS or DLS (see Results, Materials and Methods, and fig. S5). Shown are heatmaps of probability distributions of IPIs and ITIs before and after ZIP injections (5 days post-surgery recovery between pre and post). (**B**) Population results for manipulations as in (A), normalized to performance before the manipulation. Control animals received injections of retrobeads in vehicle into DLS. Left: Fraction of trials with IPI close to target (700 ms ± 20%). Right: Fraction of trials with ITI above threshold (1.2 s) (see fig. S5). (**C**) Distributions of durations between lever-presses for animals shown in (A). Pre-ZIP, last 2000 trials before ZIP; post-ZIP, first 2000 trials after ZIP. (**D**) JS divergence as a measure of dissimilarity between IPI and ITI distributions in the conditions in (A). For statistical details, see table S3 and fig. S3C for further comparison of the manipulation effects. Bars show means, dots show individual animals, and error bars show SEM. **P < 0.01.

To probe this, we took advantage of a pharmacological agent, Zeta inhibitory peptide (ZIP), which reverses plastic changes at potentiated excitatory synapses, effectively erasing locally stored memory traces (*58*). ZIP is an artificial inhibitor of the enzyme PKMzeta and likely of related kinases, which have been implicated in maintaining synaptic potentiation (*58*, *59*). While its exact mechanism of action is still subject to ongoing research, injections of ZIP lead to synaptic depotentiation and have been implicated in the degradation of various learned behaviors contingent on plasticity in the targeted structures (*58*, *60*, *61*), including the BG (*62*). In terms of learned motor behaviors, ZIP injection into motor cortex disrupts the performance of rats trained on a skilled reaching task (*56*) known to rely on plasticity in motor cortex (*57*). Thus, we can use ZIP as a tool to pinpoint circuit elements that undergo task-relevant synaptic plasticity during learning. To test whether plasticity in the DLS is essential for mastering our task, we injected ZIP into this structure in expert animals (Fig. 3 and fig. S5). We compared the outcome of ZIP injections into DLS with injections targeting either motor cortex or the DMS (Fig. 3A and fig. S5), neither of which is required for executing the learned motor sequences we train in our task (*15*, *17*) and therefore is not expected to store critical aspects of the learned behavior.

As expected, ZIP injections into the motor cortex and the DMS did not affect task performance of expert animals beyond transient effects seen in control animals undergoing sham surgeries or training breaks (Fig. 3, A to D, and figs. S4, C and D, and S2G). In contrast, ZIP injections into the DLS markedly disrupted the performance (Figs. 3, A to D, and figs. S4, C and D, and S2G), despite only reaching parts of the DLS (Materials and Methods and fig. S5B). The distinction between IPI and ITI distributions was lost (Fig. 3, B to D, and fig. S3C), and performance dropped to levels similar to the early phases of training (fig. S4, E to H). Beyond “erasing” memory traces, ZIP is not known to cause permanent changes to circuit function (*58*) and is rapidly degraded (*60*). To verify that the circuitry in the DLS remained intact, we placed the injected animals back in training and found that they relearned the task, reaching pre-ZIP performance levels [within 24,661 ± 2638 trials (means ± SD)] (fig. S4, E to I).

These results suggest that the memory of the learned movement pattern is, partly at least, formed and stored in the DLS. While our approach does not reveal the exact identity of the potentiated synapses, ZIP’s mode of action implicates excitatory synapses (*58*).

### DLS-projecting thalamic neurons are necessary for task execution

These results suggest that the DLS is necessary both for learning (see above; Fig. 2) and executing (*15*) the task-specific movement patterns we train and likely stores aspects of the motor memory (Fig. 3). This raises the question of how DLS integrates into the larger motor network to serve these functions. A recent model of how cortical and thalamic inputs to striatum contribute to generating sequential behaviors suggests that they could be stored in intrastriatal synapses (*63*). Because striatum is mostly an inhibitory structure, generating the requisite dynamics in its output neurons requires excitatory drive, which the DLS can receive from cortical, thalamic, and, for the direct pathway, nigral inputs (*3*). Our ZIP experiments, however, suggested that excitatory inputs to DLS provide more than just such a permissive drive and may function as a storage site of the motor memory (Fig. 3). Given that motor cortical input to DLS is dispensable for executing the learned behaviors (*17*) and that learning and memory formation do not require somatosensory cortex (fig. S4), we were motivated to look more closely at thalamic inputs to the DLS.

**Fig. 4.**
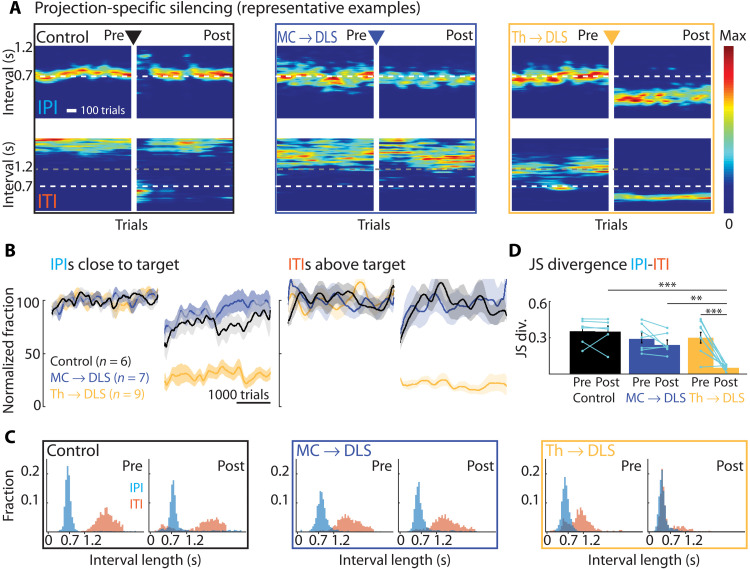
Silencing DLS-projecting thalamus but not motor cortex neurons in expert animals disrupts performance of learned motor skills. (**A**) Representative expert animals with silenced DLS-projecting neurons either in motor cortex (MC → DLS) or thalamus (Th → DLS) or control virus injections (see Results, Materials and Methods, and fig. S6). Shown are heatmaps of IPI and ITI probability distributions pre- and post-silencing (5 days post-surgery recovery between pre and post). (**B**) Population results for manipulations as in (A), normalized to performance before manipulation. Left: Fraction of trials with IPI close to target (700 ms ± 20%). Right: Fraction of trials with ITI above the threshold (1.2 s). Controls include animals expressing GFP in neurons either in motor cortex or thalamus projecting to DLS (*n* = 3 each) (see also fig. S6C). (**C**) Distributions of durations between lever-presses for animals shown in (A). Pre, last 2000 trials before silencing; post, first 2000 trials after silencing. (**D**) JS divergence as a measure of dissimilarity between IPI and ITI distributions for the same conditions as in (A). For statistical details, see table S4 and fig. S3D for further comparison of the manipulation effects. Bars show means, dots show individual animals, and error bars show SEM. **P < 0.01, ***P < 0.001.

To probe the contribution of thalamic inputs, we silenced DLS-projecting thalamus neurons in expert animals. We targeted the thalamic nuclei that provide the main source of thalamic input to the DLS, the Pf, rILN, and midline nuclei (Fig. 4A and fig. S6B) (*29*, *32*, *33*, *64*, *65*) but not motor or somatosensory thalamic nuclei. We used the same intersectional viral strategy as before (Fig. 2 and figs. S1B and S6, A and B), injecting retrogradely transported viruses for Cre expression into the DLS and AAVs for conditional TeLC expression into the thalamus (Fig. 4A and fig. S6B). As for our MC → DLS silencing experiments, the thalamic neurons we silence (DLS-projecting thalamus neurons) also send collaterals to other brain areas, most prominently to motor and somatosensory cortices (*32*, *64*–*66*). Despite this, our approach is more selective than lesions and spares populations of neurons in Pf and the rILN that project, e.g., to the DMS or to the subthalamic nucleus (*32*, *67*, *68*). As a control, we repeated the silencing of DLS-projecting motor cortical neurons (Fig. 2), but now in expert animals (Fig. 4A and fig. S6A). To further control for nonspecific effects of the surgery and viral expression, we expressed GFP in DLS-projecting neurons in motor cortex and thalamus in separate cohorts. Apart from transient effects from the break in training due to the surgical procedure and recovery time, performance in these control animals was not affected by GFP expression and the control cohorts were combined (Fig. 4, A to D, and fig. S5, C and D).

In contrast to the learning effects we had seen (Fig. 2), but consistent with results from motor cortex lesions in expert animals (*17*), silencing DLS-projecting motor cortex neurons did not impair task performance (Fig. 4, A to D, and figs. S5, C and D, and S2, D and H). Intriguingly, however, silencing DLS-projecting thalamus neurons in the Pf and rILN had very detrimental effects on task performance (Fig. 4, A to D, and figs. S5, C and D, and S2, D and H). While animals remained attentive to the task and pressed the levers during experimental sessions, consistent with the action (lever-press)–outcome (water reward) association being intact [see also (*10*, *13*)], they failed to produce the learned IPIs or ITIs (Fig. 4, A and B, and fig. S5, C and D) and lost the distinction between these two intervals altogether (Fig. 4, C and D, and fig. S3D). Notably, their post-silencing performance resembled early stages of learning (fig. S5, E to H) and did not recover even after extended periods of additional training in the task (fig. S5, E to I). This strongly suggests that DLS-projecting thalamic neurons are essential not only for executing the learned skills but also for learning them, consistent with an important role for thalamostriatal synapses in the formation of the underlying memory.

**Fig. 5.**
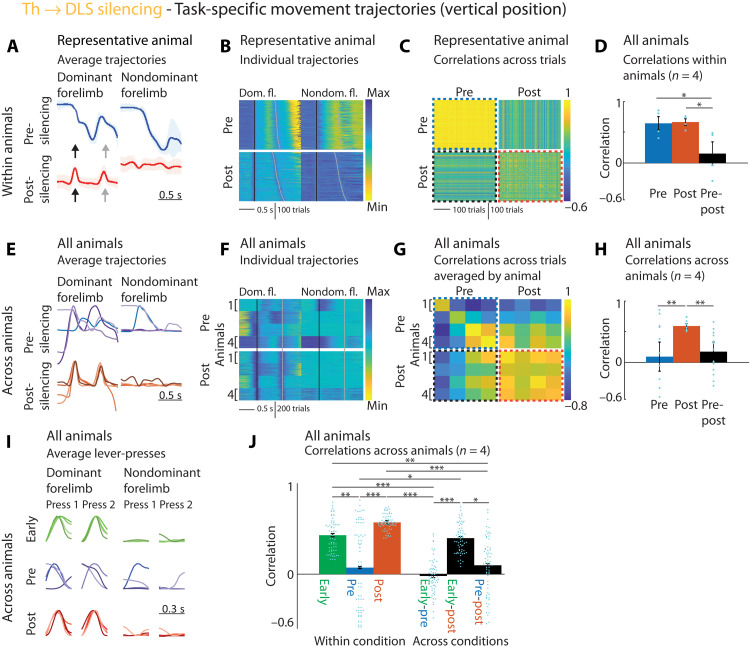
Silencing DLS-projecting thalamus neurons causes loss of idiosyncratic learned movement patterns and regression to simpler lever-pressing behaviors common across animals. (**A**) Effects of Th → DLS silencing (Fig. 4 and fig. S6) on lever-press–related forelimb movements. A representative animal’s average trajectories (vertical position) for dominant (first press) and nondominant forelimbs before/after silencing (averaged trials in range: mean IPI ± 30 ms). Arrows indicate presses. (**B**) Vertical forelimb position (color coded) of subselected trials (mean IPI ± 200 ms) of animal in (A) before/after silencing, sorted by IPI. Lines mark presses. (**C**) Pairwise correlations between trajectories [linearly time-warped (Materials and Methods)] from (B), averaged across forelimbs. Dotted lines correspond to bars in (D). (**D**) Averages of within-animal correlations [see (C)] by condition (pre-to-pre, post-to-post, and pre-to-post). Dots, individuals; bars, average across animals. (**E**) As (A), for all animals before/after silencing. (**F**) As (B), for trials (randomly selected, 150 per animal) of all animals before/after silencing. (**G**) Pairwise correlations between trials from (F), averaged across forelimbs, and per animal. Dotted lines correspond to bars in (H). (**H**) Averages of correlations in (G) by condition (pre-to-pre, post-to-post, and pre-to-post). Bars, average across animals; dots, animal-to-animal comparisons, corresponding to squares in (G). (**I**) Average trajectories during presses for all animals early in training, before and after silencing. (**J**) Averages of correlations for all presses in all trials of all animals within and across conditions in (I). Bars, average across presses for all animals; dots, individual press-to-press comparisons (average press 1 and 2 for four animals: 64 comparisons per condition). Statistics in table S5. Bar graphs: Means ± SEM. *P < 0.05, **P < 0.01, and ***P < 0.001.

While these results demonstrate that DLS-projecting thalamus neurons in the Pf and rILN are necessary for executing the learned motor skills we train, their specific function remains unclear. The performance deficits could, for example, be due to impairments in how the speed or variability of the learned movement patterns are controlled (*13*, *14*, *18*) or due to an overall inability to generate the learned movement patterns. To arbitrate between these possibilities and to gain a better understanding of the function of this pathway, we moved beyond mere measures of performance and scrutinized how the detailed kinematics of the task-related movement patterns changed after silencing these neurons. We tracked the movements of both the dominant (used for the first lever-press) and nondominant forelimbs in a subset of animals using high-speed videography and automated markerless motion tracking (Materials and Methods) (*69*, *70*). As described before (*15*, *17*), animals developed highly stereotyped and idiosyncratic movement patterns over the course of training, i.e., their forelimb trajectories were very similar across trials in the same animal (Fig. 5, A to D) but dissimilar across animals (Fig. 5, E to H). Silencing the DLS-projecting thalamus neurons markedly altered the animals’ movement patterns (Fig. 5, A to D) and essentially phenocopied our previous excitotoxic lesions of the DLS (Fig. 5) (*15*). Animals completely lost their stereotyped (Fig. 5, A to D) and idiosyncratic (Fig. 5, E to H) learned movement patterns, regressing instead to simpler, repetitive lever-pressing behaviors. The kinematic distinction between the first and second lever-press was gone, and the execution-level details of the lever-press movements were notably similar across subjects (Fig. 5, I and J), resembling the movements used early in training (Fig. 5, I to J). These results show that DLS-projecting neurons in Pf and the rILN are essential for generating the learned movement patterns we train but not required for producing species-typical lever-press movements.

## DISCUSSION

Our study was designed to elucidate how the interplay between striatum, motor cortex, and thalamus contributes to the acquisition and execution of complex task-specific movement patterns with a learned kinematic structure (Fig. 1 and fig. S7). We found that the DLS, but not the DMS, is essential for learning these skills and that this function is contingent on DLS-projecting motor cortex neurons, but not on somatosensory cortex (Fig. 2 and fig. S4). However, the very same DLS-projecting motor cortex neurons are dispensable for generating the learned movement patterns (Fig. 4), consistent with the previously published effects of less-selective motor cortex lesions (*17*). We identified plasticity at excitatory synapses in DLS as a likely substrate for the underlying motor memory (Fig. 3) and further showed that DLS-projecting thalamic neurons (in the rILN and Pf) are essential for executing the consolidated behaviors (Figs. 4 and 5) and that their loss prevents relearning of the task (fig. S5, E to I). While further mechanistic studies at the synapse level will be necessary, the results presented here are consistent with motor cortical inputs to DLS guiding plasticity at thalamostriatal synapses, thus allowing subcortical motor circuits to learn and execute stereotyped task-specific movement patterns (fig. S7). Together, these findings shed new light on the neural circuit-level logic by which motor skills with learned task-specific kinematic movement patterns are acquired, specifically the roles of two of DLS’s major inputs, from motor cortex and thalamus (fig. S7).

### Role of the thalamostriatal pathway in motor skill execution

To date, the role of thalamic inputs to the striatum from Pf and the rILN has mostly been studied and discussed in terms of how they modulate signal flow and plasticity at corticostriatal synapses and how they contribute to attention and behavioral flexibility (*20*, *23*, *33*, *42*, *43*). Experimental studies have been consistent with these thalamic nuclei providing a state and/or context-related signal that allows associations between the environment and appropriate movements and actions to be learned (*23*, *42*, *43*). While more detailed studies will be required, our results substantially inform our understanding of this thalamostriatal pathway and its function by implicating DLS-projecting thalamus neurons both in the control of learned complex task-specific movement patterns and in the storage of the underlying motor memories. This is consistent with previous findings describing a role of other thalamostriatal pathways (Pf to DMS and VP to DLS) in the initiation and execution of action sequences (*38*). However, an important open question is what role the cortical collaterals of these neurons (*32*, *64*–*66*) play and whether and to what degree they contribute to these processes. Furthermore, our experiments did not address whether the nonhomogeneous thalamus inputs have distinct functions in this process (*33*). Inputs originating from Pf and the rILN differ in striatal projection patterns (broad versus focused arborization and targeting of dendritic shafts versus spines) (*71*) and physiological characteristics such as short-term plasticity (*72*). These differences and the different information the individual inputs may transmit could indicate distinct contributions to both learning and execution of new motor skills and to memory formation and storage. Building on the findings presented here, future studies are warranted to disentangle the functions of the individual thalamic nuclei by selectively manipulating their striatal projections and their collaterals.

### Motor cortical tutoring extends subcortical functionality

Our study also sheds new light on the roles of the motor cortex and its subcortical targets. Many prior studies on motor learning have either used motor cortex–dependent dexterous tasks (*25*, *26*) or assumed that motor cortex controls the acquired behavior (*2*, *24*), making it difficult to distinguish its separate contributions to learning and control processes and to parse the independent control functions of subcortical motor circuits. In contrast, the execution of the highly stereotyped task-specific movement patterns we train is motor cortex independent (*17*), suggesting that its control is largely subcortical (*15*, *17*). The involvement of both the BG (*15*) and the thalamus (Figs. 4 and 5) in the execution of the learned movement patterns implicates the phylogenetically older subcortical pathway, which connects thalamus and the BG to subcortical motor control centers (*4*, *7*). It is widely assumed that this subcortical pathway is involved in initiating and modulating (*1*, *7*) innate behaviors, such as locomotion, grooming, and feeding (*73*, *74*) through selective and graded disinhibition of its downstream targets. Our results suggest that this BG pathway can be recruited to support the generation of novel task-specific movement patterns, a process that may require a motor cortex–dependent reprogramming of subcortical motor circuits. This would allow the cortex to off-load the “task” of generating specialized and stereotyped learned behaviors to subcortical circuits, making them more automatized and less prone to cortical “interference,” and hence perhaps more robust (*75*). Consistent with this idea are observations that task-related cortical activity decreases over the course of training and with increasing automaticity (*22*, *76*). Our current study suggests that the striatum may be where information from cortex is “handed-off” to subcortical circuits. Many open questions remain regarding how this process is implemented in the interplay between the striatum and its cortical and thalamic inputs. For example, it remains unclear whether plastic changes in motor cortex or at corticostriatal synapses occur early in learning and enable the formation of the final, motor cortex–independent memories.

The technical limitations of our approach did not allow us to probe whether the distinct motor cortical projections, originating from intratelencephalic or pyramidal tract neurons (*77*), play differential roles in this process (*78*). Similarly, it remains an open question if and how the collaterals to other brain areas (e.g., other cortices, thalamus or pons) of the DLS-projecting motor cortex neurons (*29*, *48*), which were also silenced in our experiments, contribute to learning the task-specific movement patterns. Properly disentangling this question will require studies that silence axonal projections of these neurons in specific brain regions (*79*).

## MATERIALS AND METHODS

### Animals

The care and experimental manipulation of all animals were reviewed and approved by the Harvard Institutional Animal Care and Use Committee. Experimental subjects were female Long Evans rats 3 to 10 months old at the start of training (*n* = 77; Charles River). Because the behavioral effects of our circuit manipulations could not be prespecified before the experiments, we chose sample sizes that would allow for identification of outliers and for validation of experimental reproducibility. Animals were excluded from experiments post hoc if the lesions or infected areas were found to be outside of the intended target area or extended into additional brain structures (see the “Quantification of lesion size, viral infection, and ZIP spread” section). The investigators were not blinded to the allocation during experiments and outcome assessment, unless otherwise stated.

### Behavioral training

#### Initial lever-press training

To familiarize the rats with the behavioral setup and to train them to press the lever to receive a reward, they underwent a few initial training stages, which collectively lasted 1 to 2 days (*17*). First, rats were conditioned to collect reward from a water spout in response to a reward tone (4 kHz of pure tone; 250 ms). Next, pressing the lever triggered the reward tone and reward delivery. After 50 rewarded lever-presses, reward probability was lowered to 30%. After 50 additional rewarded lever-presses, animals transitioned to the timed lever-press protocol described below.

#### Timed lever-press sequence training

After the initial training, the rats were trained in a lever-pressing task as previously described (*17*). Water-restricted animals were rewarded with water for pressing a lever twice within performance-dependent boundaries around a prescribed interval between the presses (IPI = 700 ms). The reward boundaries were initially set at 200 and 1100 ms and dynamically and automatically adjusted to shape the animals’ behavior toward the target while maintaining reward rates between 30 and 40%. Reward boundaries were automatically updated between sessions, tightening if the reward rate was more than 40% and relaxing if it was less than 30%. To further incentivize IPIs close to the target, a reward landscape [see (*17*)] with five evenly distributed levels was established between the target (largest reward) and each reward boundary (smallest reward). All adjustments to the reward boundaries and landscapes were done automatically, purely based on the animals’ performance without any manual intervention. In addition, animals had to withhold pressing for 1.2 s after unsuccessful trials before initiating a new trial (ITI). All animals were trained in a fully automated home cage training system (*17*). Manipulations were either performed in naïve animals before the beginning of the training (Fig. 2) or after they had reached our learning criteria (mean IPI = 700 ms ± 10%; CV of IPI distribution < 0.25; see the “Behavioral data analysis” section below) and a median ITI > 1.2 s, indicating that they had learned the task structure and stabilized their performance (Figs. 3 to 5).

### Striatal lesion surgeries

Bilateral striatal lesions in naïve animals, targeting either the motor cortex–recipient part (DLS) or the non-MC input–receiving part (DMS), were performed in two stages with a 10-day break between surgeries. Lesions were performed as previously described (*17*). Briefly, animals were anesthetized with 2% isoflurane in carbogen and placed in a stereotactic frame. After incision of the skin along the midline and cleaning of the skull, bregma was located and small craniotomies for injections were performed above the targeted brain areas (for injection coordinates see Table 1). A thin glass pipette connected to a microinjector (Nanoject II, Drummond) was lowered to the injection site, and the excitotoxin quinolinic acid [0.09 M in phosphate-buffered saline (PBS) (pH 7.3); Sigma-Aldrich] was injected in 4.9-nl increments to a total volume of 175 nl per injection site, at a speed of <0.1 μl/min. After injection, the glass pipette was retracted by 100 μm and remained there for at least 3 min before further retraction to allow for diffusion and to prevent backflow of the drug. After all injections were performed, the skin was sutured and animals received painkillers (buprenorphine, Patterson Veterinary). Animals were allowed to recover for 10 days after the second surgery before being put into training.

**Table 1.** Injection coordinates for striatal lesions. Coordinates are given in millimeters. AP (anterior/posterior) and ML (medial/lateral) coordinates are measured from bregma and DV (dorso/ventral) from the brain surface.

	**DLS**				**DMS**			
AP	+0.7	+0.7	−0.3	−0.3	+1.2	+1.2	+0.2	+0.2
ML	±3.6	±3.6	±4.0	±4.0	±1.9	±1.9	±1.9	±1.9
DV	−5.5	−4.5	−5	−4	−5.5	−4.5	−5.5	−4.5

To test for nonspecific effects of surgery and striatal injections on behavior, we performed one-stage control surgeries according to the procedure described above, bilaterally injecting fluorophore-coated latex microspheres [red excitation (exc.) = 530 nm, emission (em.) = 590 nm; and green exc. = 460 nm, em. = 505 nm] referred to as retrobeads (Lumafluor), diluted 1:100 in PBS into DLS. This allowed for post hoc evaluation of the targeting of our control injections. This control group was combined in Fig. 2 with a group of animals injected with viruses in DLS and motor cortex for expression of GFP in DLS-projecting motor cortex neurons (see the “Projection-specific silencing of synaptic transmission” section below for details). The two groups (*n* = 3 each) are shown separately in fig. S1F.

### Somatosensory cortex lesion surgeries

Bilateral lesions of somatosensory cortex in naïve animals (for injection coordinates, see Table 2) were performed as motor cortex lesions previously described in (*17*) and the striatal lesions described above. Different from the procedure above, lesions were induced by incremental injection of the excitotoxic agent ibotenic acid (1%) to a total volume of 115 nl per injection site.

**Table 2.** Injection coordinates for somatosensory cortex lesions. Coordinates are given in millimeters. AP and ML coordinates are measured from bregma.

	**S1**					
AP	+0	+0	−1.8	−1.8	−1.8	−1.8
ML	±4.5	±4.5	±4.5	±4.5	±2.5	±2.5
DV	−1.6	−0.8	−1.6	−0.8	−1.6	−0.8

### ZIP injections

To inhibit the enzyme PKMzeta and to reverse synaptic plasticity in different target regions, we injected the inhibitory peptide ZIP (*58*) into either motor cortex, DLS, or DMS (for injection coordinates, see Table 3). Animals were injected once they had reached our learning criteria (see the “Behavioral training” section). We performed one-stage injection surgeries according to the procedure described above. ZIP (10 mM in PBS; Tocris) was injected in 10-nl increments to a total volume of 500 nl per injection site (*56*). While the concentration of ZIP used here is on the higher end of previously used concentrations and may potentially affect additional enzymes and not exclusively PKMzeta (*58*, *59*), it has been shown that injections of ZIP at this concentration lead to memory erasure (*56*). We would like to emphasize that ZIP is used here as a tool to determine whether memories are stored in a certain brain area and not to determine the mechanisms of how ZIP affects memory storage. To achieve a wider spread of the injected drug, injections were performed over a dorsal-ventral range by slowly moving the injection pipette dorsally while continuously injecting at evenly spaced intervals. Injection sites were verified post hoc by locating co-injected fluorescent (retro-)beads. Control animals were injected in the same way in the DLS but only with retrobeads. No animals had to be excluded based on the injection sites. Animals were put back into training after 5 days of recovery.

**Table 3.** Injection coordinates for ZIP injections. Coordinates are given in millimeters. AP and ML coordinates are measured from bregma.

	**MC**					**DMS**			**DLS**			
AP	+1	+1	+1.5	+2.25	+3	+1.5	+1	+0.2	+1.75	+1	−0.25	−0.7
ML	±2	±4	±2.75	±2.5	±2	±1.9	±1.9	±1.9	±3.2	±4	±4	±4
DV	−1.5 to −0.7	−1.5 to −0.7	−1.5 to −0.7	−1.5 to −0.7	−1.5 to −0.7	−5 to −3.4	−5.8 to −4	−5.8 to −3.8	−4.8 to −3.5	−4.5 to −3.5	−5 to −3.5	−5 to −3.8

To estimate the spread of ZIP in the different brain areas, we injected a separate cohort of animals with ZIP-Biotin (10 mM in PBS; Tocris) (*56*), which could be visualized post hoc. We note that ZIP-Biotin has a higher molecular weight than uncoupled ZIP and is therefore expected to diffuse less far, providing only a lower bound for the actual spread of uncoupled ZIP in our experimental animals. Furthermore, the time course of the diffusion of uncoupled ZIP and the time of the biggest extent of its spread are unclear. We therefore determined the spread of ZIP-Biotin at two time points. Animals were perfused either 2 hours after ZIP injection (*n* = 1 each for MC, DMS, and DLS) or 24 hours after injection (*n* = 1, DLS). We used either fluorescein isothiocyanate–coupled avidin [1:100 in blocking solution (see the “Histology” section); Thermo Fisher Scientific] or fluorescently labeled anti-biotin antibodies [1:400 in blocking solution (see the “Histology” section); Jackson ImmunoResearch) for visualization of alternate slices of the individual brains (see the “Histology” section).

### Projection-specific silencing of synaptic transmission

To silence neurons either in motor cortex or in thalamus that send axons to the DLS, we used a two-component viral strategy (for injection coordinates, see Table 4). We injected viruses, which can retrogradely infect neurons by entering axon terminals, into the DLS [either CAV (*45*) (Institut de Genetique Moleculaire de Montpellier, Montpellier) or rAAV (*46*) (Janelia, Addgene)]. These viruses drive the expression of Cre recombinase in infected neurons. We further injected AAVs for Cre-dependent conditional expression of TeLC coupled to GFP (TeLC-GFP) (*47*) [DNA construct shared by P. Wulff (University of Kiel), custom virus production by Harvard Medical School, Ocular Genomics Institute, Gene Transfer Core] into either motor cortex or thalamus. While the AAVs nonspecifically infect neurons in the target area, TeLC is only expressed in neurons expressing Cre, i.e., neurons infected by the retrograde viruses. This strategy allowed us to target cortical or thalamic projection neurons, which send an axon to the DLS. We note, however, that this approach does not exclusively target striatal projections, because these neurons also send collaterals to other brain areas, including different cortices, thalamus or pons for motor cortical neurons (*29*, *48*), and sensorimotor cortices for thalamic neurons (*32*, *64*–*66*). Nevertheless, this approach spares the local circuitry and large populations of projection neurons that do not target the DLS [see, e.g., (*29*, *32*, *50*, *51*, *68*)]. TeLC cleaves the synaptic protein VAMP2, thereby preventing the fusion of synaptic vesicles with the membrane and the release of neurotransmitters, effectively silencing synaptic transmission in infected neurons. Injections were performed as one-stage surgeries, and virus was injected in 10-nl increments to a total volume of 300 nl (MC), 400 nl (DLS), and 200 nl (thalamus) per injection site. Surgeries were performed either in naïve (Fig. 2) or expert animals (Figs. 4 and 5), and they were given 5 days for recovery from surgery before the (re-)start of training. Spread of the TeLC expression was determined post hoc (see the “Histology” section). To control for the effects of surgeries, viral infections, and expression of recombinant proteins, we performed the same injections but using an AAV for expression of GFP (Penn Vector Core) instead of TeLC-GFP (MC → DLS for Fig. 2; MC → DLS and Th → DLS for Fig. 4).

**Table 4.** Injection coordinates for projection-specific silencing. Coordinates are given in millimeters. AP and ML coordinates are measured from bregma.

	**MC**					**DLS**			
AP	+1	+1	+1.5	+2.25	+3	+1.75	+1	−0.25	−0.7
ML	±2	±4	±2.75	±2.5	±2	±3.2	±4	±4	±4
DV	−1.5 to −0.7	−1.5 to −0.7	−1.5 to −0.7	−1.5 to −0.7	−1.5 to −0.7	−4.8 to −3.5	−4.5 to −3.5	−5 to −3.5	−5 to −3.8
	**Thalamus**								
AP	−3.3		−3.3		−3.8	−3.8		−4.2	
ML	±1.2		±0.4		±1.4	±0.4		±1.4	
DV	−5.8 to −4.3		−6.2 to −5.8		−6.2 to −4.3	−6.1 to −5.9		−6.1 to −4.8	

Because even low levels of TeLC expression may lead to silencing of infected neurons, we tested whether our results could be due to unspecific Cre-independent leaky expression of TeLC in non-projection neurons in targeted areas. To verify the specificity of our silencing approach, we injected a separate cohort of naïve animals only with the TeLC AAV in motor cortex and not with the retrograde viruses for Cre expression and tested their ability to learn our task (fig. S1, E and F). We reasoned that if the observed effects of silencing on behavior were due to unspecific Cre-independent expression of TeLC, they should be recapitulated by this control manipulation.

To estimate the infection rate of our viral approach, we co-injected retrobeads (Lumafluor) (see the “Striatal lesion surgeries” section above) into DLS in a subset of animals. Retrobeads are taken up by axonal terminals and transported retrogradely with high efficiency, allowing us to use the number of retrobead-labeled neurons in cortex or thalamus as an estimate for the number of neurons projecting to DLS from these areas. We determined the efficiency of our viral approach by comparing the numbers of retrobead-labeled and GFP-expressing neurons at the injection sites in the cortex or thalamus. We counted neurons in regions of interest in motor cortex or thalamus in three slices per animal (*n* = 2 animals each for cortex and thalamus). Infection rates were similar between cortex and thalamus and reached about 50% of retrobead-labeled neurons at the centers of the injection sites (motor cortex: 56 and 53%; thalamus: 71 and 38%).

### Histology

At the end of the experiment, animals were euthanized [ketamine (100 mg/kg) and xylazine (10 mg/kg); Patterson Veterinary), perfused with 4% paraformaldehyde, and their brains were harvested for histology to confirm the lesion size, injection sites, and drug/viral spread. The brains were sectioned into 80-μm slices and stained appropriately. To determine lesion location and size, slices were stained with Cresyl Violet following standard procedures. In a subset of animals, immunofluorescence staining was performed instead of Cresyl Violet staining. After slicing, sections were blocked for 1 hour at room temperature in blocking solution (1% bovine serum albumin and 0.3% Triton X-100), stained overnight at 4°C with primary antibodies for NeuN (1:500 in blocking solution; to stain for neuronal cell bodies; Millipore, MAB377) and glial fibrillary acidic protein (1:500 in blocking solution; to stain for glia cells; Sigma-Aldrich, G9269), and subsequently with appropriate fluorescently coupled secondary antibodies (1:1000 in blocking solution) for 2 hours at room temperature. The same staining protocol was used to visualize TeLC-GFP, using antibodies for NeuN and GFP (1:1000 in blocking solution; Life Technologies, A11122), or to visualize ZIP-Biotin, using antibodies for biotin (1:400 in blocking solution; Jackson ImmunoResearch, 200-002-211). In alternate slices, ZIP-Biotin was visualized by incubation with fluorescein-coupled avidin (1:100 in blocking solution; Thermo Fisher Scientific) overnight. Images of whole brain slices were acquired at ×10 magnification with either the VS210 Whole Slide Scanner (Olympus) or the Axioscan Slide Scanner (Zeiss).

### Quantification of lesion size, viral infection, and ZIP spread

To determine the extent and location of striatal lesions, we analyzed several sections (*4*–*6*) spanning the anterior-posterior extent of the striatum, allowing for an estimate of the overall lesion size. Lesion boundaries were determined throughout the striatum and adjacent areas, blind to the animals’ identity and performance. Boundaries were marked manually based on differences in cell morphology and density (loss of larger neuronal somata and accumulation of smaller glial cells). The extent of the striatum was determined on the basis of the Paxinos Rat Brain Atlas, using anatomical landmarks (external capsule and ventricle) and cell morphology and density. In addition, we marked the globus pallidus externus (GPe) in posterior sections because mistargeted injections may lead to its partial lesioning, disrupting the output both of the DLS and DMS.

In addition to the overall lesion size, we also determined the lesioned fractions of the DLS/DMS. Because the DLS and DMS are not clearly defined, we made use of their differential input patterns from MC and PFC, respectively, which we had previously determined using viral anterograde labeling (*15*). We used these identified boundaries of the DLS and DMS to determine the lesioned fractions in the experimental animals. We predefined a threshold based on our previous observations (*15*) of at least 50% loss of the targeted region, less than 10% loss of the nontargeted part of the striatum, and no lesions in the GPe for inclusion of experimental animals in our analysis. On the basis of this threshold, no animals had to be excluded.

To determine the spread of TeLC expression in our silencing experiments, we determined the affected areas in motor cortex and thalamus. We manually labeled the extent of the infections based on the presence of GFP-expressing somata in the respective regions. Animals with no discernable expression of GFP in cortex or in thalamus were excluded from the behavioral analysis (*n* = 2 and *n* = 1, respectively). We further used co-injected fluorescent beads to verify the injection sites of the retrograde viruses in the DLS.

To determine a lower boundary for the spread of ZIP injections in the different brain areas, we manually labeled the extent of fluorescent labeling around the injection sites in the cohort of animals injected with ZIP-Biotin. In experimental animals injected with nonlabeled ZIP, we used co-injections of retrobeads to verify the injection sites in the respective target areas. On the basis of this, no animals had to be excluded.

### Kinematic tracking

To determine the movement trajectories of the forelimbs of animals performing our task, we made use of recently developed machine learning approaches, using deep neuronal networks to determine the position of specific body parts in individual video frames (*69*, *70*). Videos of animals performing the task were acquired at 30 Hz and saved as snippets ranging from 1 s before the first lever-press in a trial to 2 s after the last lever-press in the trial. We extracted about 1000 frames randomly selected throughout the duration of the trials, balanced across pre- and post-manipulation conditions, and manually labeled the position of the forelimbs in each frame using a custom-written MATLAB code. These data were used to train individual neural networks for each animal.

We trained ResNet-50 networks that were pretrained on ImageNet, using DeeperCut (https://github.com/eldar/pose-tensorflow) (*69*). Training was performed using default parameters (1 million training iterations, three color channels, with pairwise terms, and without intermediate supervision). Data augmentation was performed during training by rescaling images from a range of 85 to 115%.

The trained neural network was then used to predict the position of the forelimbs in all trials across conditions, on a frame-by-frame basis. The position of a forelimb in a frame is given by the peak of the network’s output score map. Frames in which the forelimb was occluded were identified as having a low peak score. For both the training and the subsequent predictions, we used GPUs in the Harvard Research Computing cluster.

Because the two forelimbs could often be confused for each other in the neural network’s predictions from a single frame, we took advantage of correlations across time to constrain the predictions. For each forelimb, the predicted score maps for all frames in a single trial video were passed through a Kalman filter using the Python toolbox filterpy. Specifically, a constant-acceleration Kalman smoother was used, which assumes that the forelimb on adjacent frames will have the same acceleration (zero jerk) plus a small noise term. Only frames with a weak neural network prediction score were adjusted by the Kalman filter; otherwise, the original neural network prediction was used as the forelimb position.

The tracking accuracy was validated post hoc by visual inspection of at least 50 predicted trajectories per animals. Initial training with lower frame numbers often led to inaccurate tracking results. After settling on 1000 training frames, none of the trained networks was discarded.

Missing frames in the trajectories, e.g., due to temporary occlusions of the forelimbs, were linearly interpolated for a maximum of five consecutive frames. If occlusions lasted longer, then the trajectories were discarded. In a subset of animals, the quality of the recorded videos was not sufficient for high-quality tracking of the forelimbs, due to inappropriate lighting conditions or due to occlusions of the forelimbs over long durations of the trials, and we had to discard the trajectories (*n* = 4).

### Behavioral data analysis

#### Performance metrics

Performance metrics were determined on the basis of the timing of lever-presses in our task. The IPI was determined as the time between the first and second press in a trial, and the ITI was determined as the time between the last press in an unsuccessful trial and the next occurring lever-press. The CV during learning (Fig. 2 and fig. S1) was calculated across 100 trials, and the moving average was low-pass–filtered with a 300-trial boxcar filter. For the manipulations in expert animals (Figs. 3 and 4 and figs. S3C and S4C), a smaller moving window (25 trials) and boxcar filter (50 trials) were used. The fraction of trials close to the target IPI was calculated using the same windows and filters. Trials were labeled as close to the target if they were in the IPI range of 700 ms ± 20%.

#### Criterion performance

We considered animals as having successfully learned the task and as having reached criterion performance if the CV was less than 0.25 and the mean of the IPI distribution was in the range of 700 ms ± 10% for a 3000-trial sliding window. These criteria were previously established and confirmed to capture the learning performance of intact animals in our task (*17*). Furthermore, these criteria can discriminate between intact animals that learn and animals that had undergone manipulations and show impaired learning (*17*).

#### Jensen-Shannon divergence

As a measure for the dissimilarity of the IPI and ITI distributions in individual animals, we calculated the Jensen-Shannon divergence (JSD) of the distributions. The JSD is a symmetric derivative of the Kullback-Leibler divergence (KLD). We calculated the JSD as

JSD(IPI∣∣ITI)=12KLDIPI(IPI∣∣M)+12KLDITI(ITI∣∣M)

where *M* = (IPI + ITI)/2

$${\text{KLD}}_{\text{IPI}}=\sum \text{IPI}\;\text{log}(\frac{\text{IPI}}{M})$$

$${\text{KLD}}_{\text{ITI}}=\sum \text{ITI}\;\text{log}(\frac{\text{ITI}}{M})$$

#### Identification of sequence modes

Close examination of task-related kinematics revealed that individual rats often solve the interval pressing task using multiple unique but related motor sequences that we refer to as “sequence modes” [for details about sequence modes and their identification and analysis, see (*15*)]. To systematically identify these sequence modes, we performed unsupervised clustering of all task-associated kinematics recorded from individual rats. These kinematic data included the horizontal and vertical components of position, velocity, and acceleration of both forelimbs, recorded during a period ranging from 200 ms preceding the first lever-press to 200 ms following the second lever-press, for all trials in which the rat performed at least two lever-presses within 1.2 s. To account for trial-to-trial variability in IPIs, we linearly time-warped (i.e., resampled) kinematic data recorded between the two lever-presses to a target interval of 700 ms. The variance of each kinematic feature (position, velocity, or acceleration) was standardized by dividing by its SD estimated across all time points and horizontal and vertical components for both forelimbs. After preprocessing, we performed two-dimensional *t*-distributed stochastic neighborhood embedding of the kinematic data associated with each trial (all kinematic features and components, forelimbs, and time points). We then applied density peak clustering (*80*) to identify putative sequence modes. In the final step, we corrected for overclustering by the density peak algorithm by examining the task-aligned kinematic traces for each cluster and combining those which had very similar kinematics.

#### Movement trajectory analysis

We compared the trajectories of both forelimbs of all tracked animals before and after Th-DLS silencing (Fig. 5). We focused on the position of the forelimbs in the vertical dimension, in which the movements in our task are more pronounced than in the horizontal dimension. To be able to compare the stereotypy of the trajectories for the learned motor sequences, we subselected trials that were successful and rewarded and that occurred after unrewarded trials. This allowed us to compare trials with the same start and end positions. This is necessary because animals move down to, and back up from, a reward port underneath the lever after successful trials (*17*). Some animals learn more than one stereotyped motor sequence as solutions to our task. These solutions, to which we refer as “modes,” occur at different frequencies, usually with one mode being predominant, but all become stereotyped and have similar properties (see the “Identification of sequence modes” section above). For our analysis, we subselected only trials from the most common mode so that trajectories are comparable. We plotted the average of the selected trajectories for before and after the manipulation, calculated the SEM (Fig. 5A), and plotted a projection of all selected trials (Fig. 5B). To calculate the correlations between the individual trials, we linearly warped the trajectories to the same duration by interpolating between the lever-presses. Because the lever-presses themselves have stereotyped trajectories, largely independent of the trial duration, we interpolated only the trajectories from 100 ms after the first to 100 ms before the second lever-press to preserve the shape of the presses. From these warped trajectories, we calculated trial-to-trial correlations separately for both forelimbs and averaged the correlations for each trial (Fig. 5C). These correlations were averaged for the individual conditions within animals, and those means were averaged across animals and plotted with the SEM (Fig. 5D).

To compare the trajectories across animals, we linearly warped all trajectories and normalized their amplitude to their individual maximum amplitude (Fig. 5, E and F). To calculate the correlations across animals, we first calculated the average pairwise correlations across all trials within individual animals and then averaged these across the individual animals (Fig. 5, G and H).

We separately compared the lever-press movements, defined as the trajectory in the range of ±150 ms around a detected lever-press (Fig. 5, I and J). We normalized the lever-press trajectories to their individual maximum amplitude and plotted their overlay (Fig. 5I). To compare the lever-presses before and after the manipulation to the presses early in training, we additionally subselected trials as described above from the first 2000 trials of training. As above, we calculated the average pairwise correlations for all lever-presses in all trials of all animals across the conditions (early, pre-, and post-silencing) and averaged them first by lever-press (i.e., animal 1 press 1, animal 1 press 2, etc.) and then by condition (Fig. 5J).

## Notes

View/request a protocol for this paper from *Bio-protocol*.
