# Supplementary material for: Distinct roles for motor cortical and thalamic inputs to striatum during motor skill learning and execution
Source: Sci Adv. 2022 Feb 25;8(8):eabk0231. doi: 10.1126/sciadv.abk0231 (PMC8880788; doi:10.1126/sciadv.abk0231)
Supplement: Supplementary file 1 — Figs. S1 to S7 Table S1 to S5 [file sciadv.abk0231_sm.pdf]

Science Advances
AAAS

Supplementary Materials for

Distinct roles for motor cortical and thalamic inputs to striatum during motor skill learning and execution

Steffen B. E. Wolff*, Raymond Ko, Bence P. Ölveczky*

*Corresponding author. Email: olveczky@fas.harvard.edu (B.P.Ö.); swolff@som.umaryland.edu (S.B.E.W.)

Published 25 February 2022, *Sci. Adv.* **8**, eabk0231 (2022)
DOI: 10.1126/sciadv.abk0231

The PDF file includes:

Figs. S1 to S7
Table S1 to S5
Legend for movie S1

Other Supplementary Material for this manuscript includes the following:

Movie S1

Supplementary Figure 1

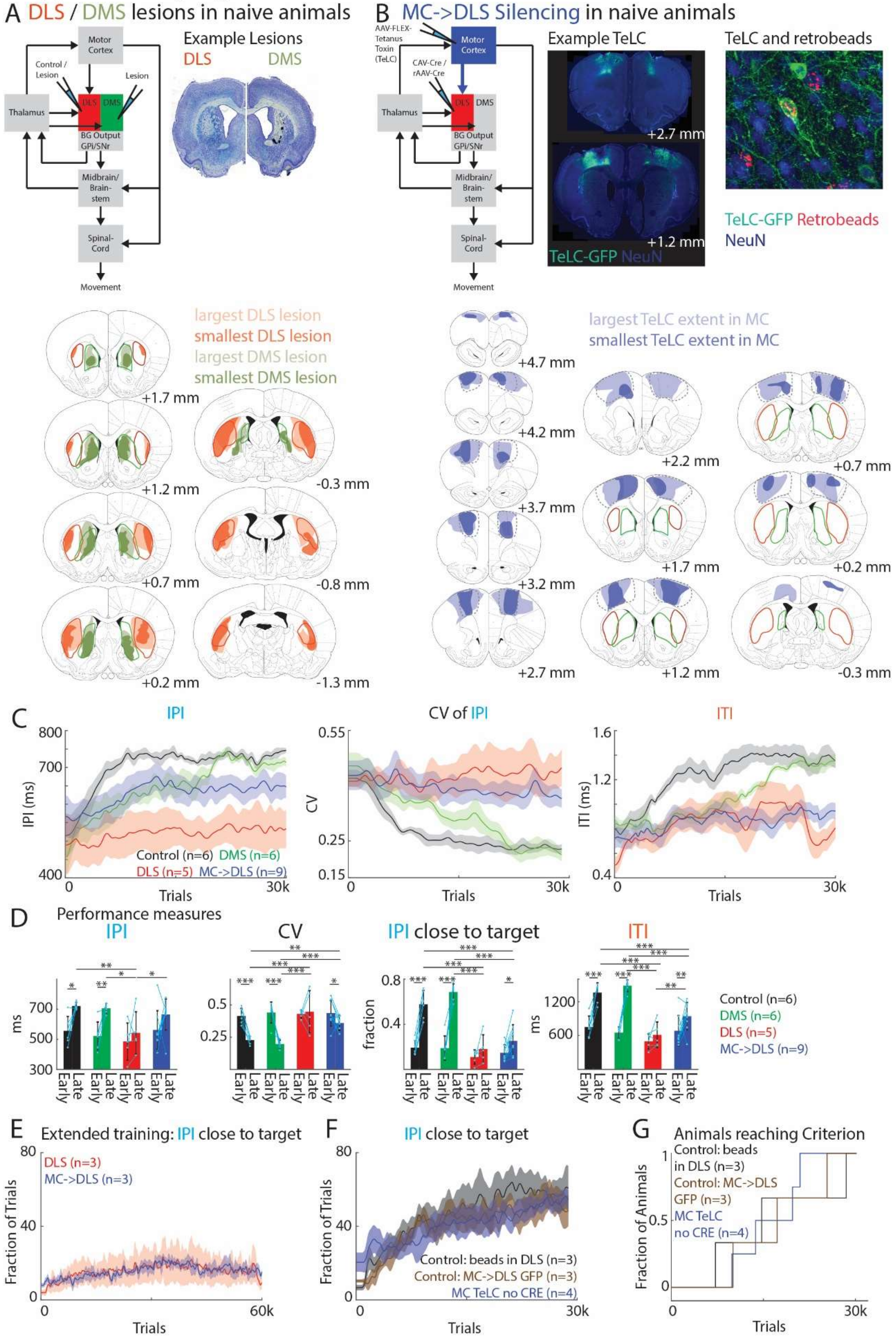

Fig. S1.

Extent of lesions of the DLS and DMS and of TeLC expression in motor cortex and resulting behavioral effects. A) *Top left:* Experimental scheme for excitotoxic lesions (see Fig. 2). *Top right*: Example lesions of DLS and DMS, respectively. *Bottom*: Red/green: Extent of DLS/DMS lesions. Light and dark colors indicate the extent of the largest and smallest lesion, respectively. Red and green outlines mark the extent of motor and prefrontal cortex projections to the striatum as previously identified by viral anterograde labeling of projection fibers (*16*). **B)** *Top left:* Experimental scheme for projection-specific silencing (see Fig. 2). Note, that this approach also silences the collaterals to other brain areas of the targeted DLS-projecting motor cortex neurons. *Top right*: Example TeLC-GFP expression in motor cortex and close up of neurons labeled with retrobeads and expressing TeLC-GFP. *Bottom*: Light and dark blue: largest and smallest extent of TeLC-GFP expression, respectively. Grey outlines mark the extent of the area in motor cortex which was targeted for silencing, based on our previous lesions of motor cortex (*16*, *24*). **C)** Population results for performance measures over the course of learning after different manipulations (as in Fig. 2A). IPI: inter-press interval, CV of IPI: Coefficient of Variation of the IPI, ITI: inter-trial interval. See Table S1 for a statistical comparison between the learning curves. **D)** Comparisons of performance measures between different manipulations early and late in training (as in Fig. 2). IPI: inter-press interval, CV: Coefficient of Variation of the IPI, IPI close to target: Fraction of trials close to target IPI (700 ms +/- 20%), ITI: inter-trial interval. Early: First 2000 trials in training, Late: trials 30,000 to 32,000 in training. Bars represent means across animals and dots represent means within individual animals. Error bars represent standard error of the mean (SEM). Two-way repeated measures ANOVAs (mixed effects model) were conducted for all performance measures. For the IPI significant effects of manipulation, (F(3, 22) = 3.60, p=0.03) and of time point (early vs. late) (F(1, 22) = 17.50, p<0.001), but no significant interaction between manipulation and time point, (F(3, 22) = 0.85, p=0.48) were found. Simple main effects analysis showed a significant difference between the early and late time points for Control (p=0.014) and DMS animals (p=0.007), but no differences for DLS (p=0.41) and MC->DLS (p=0.054) animals. There were no differences between manipulations in the early time point (Control vs. DMS p=0.55; Control vs. DLS p=0.26; Control vs. MC->DLS p=0.92; DMS vs. DLS p=0.57; DMS vs. MC->DLS p=0.45; DLS vs. MC->DLS p=0.19). In the late time point DLS animals were different from all others (vs. Control (p=0.006); vs. DMS (p=0.011); vs. MC->DLS (p=0.036), but there were no differences between the other groups (Control vs. DMS (p=0.79); Control vs. MC->DLS (p=0.297); DMS vs. MC->DLS (p=0.448)). For the CV significant effects of manipulation, (F(3, 22) = 6.19, p=0.003) and of time point (early vs. late) (F(1, 22) = 31.16, p<0.001) and a significant interaction between manipulation and time point, (F(3, 22) = 6.45, p=0.003) were found. Simple main effects analysis showed a significant difference between the early and late time points for Control, DMS (both p<0.001) and MC->DLS animals (p=0.048), but not for DLS (p=0.72) animals. There were no differences between manipulations in the early time point (Control vs. DMS p=0.551; Control vs. DLS p=0.734; Control vs. MC->DLS p=0.593; DMS vs. DLS p=0.819; DMS vs. MC->DLS p=0.905; DLS vs. MC->DLS p=0.891). In the late time point DLS and MC->DLS animals were different from Control (p<0.001 and p=0.003) and DMS (both p<0.001) animals, but not from each other (p=0.053). There were no differences between Control and DMS (p=0.516). For the trials with IPI close to target significant effects of manipulation, (F(3, 22) = 18.81, p<0.001) and of time point (early vs. late) (F(1, 22) = 105.29, p<0.001) and a significant interaction between manipulation and time point, (F(3, 22) = 16.55, p<0.001) were found. Simple main effects analysis showed a significant difference between the

early and late time points for Control, DMS (both p<0.001) and MC->DLS animals (p=0.021), but not for DLS (p=0.229) animals. There were no differences between manipulations in the early time point (Control vs. DMS p=0.947; Control vs. DLS p=0.191; Control vs. MC->DLS p=0.398; DMS vs. DLS p=0.213; DMS vs. MC->DLS p=0.439; DLS vs. MC->DLS p=0.529). In the late time point DLS and MC->DLS animals were different from Control (both p<0.001) and DMS (both p<0.001) animals, but not from each other (p=0.219). There were no differences between Control and DMS (p=0.072). For the ITI significant effects of manipulation, (F(3, 22) = 14.53, p<0.001) and of time point (early vs. late) (F(1, 22) = 70.08, p<0.001) and a significant interaction between manipulation and time point, (F(3, 22) = 8.81, p<0.001) were found. Simple main effects analysis showed a significant difference between the early and late time points for Control, DMS (both p<0.001) and MC->DLS animals (p=0.008), but not for DLS (p=0.357) animals. There were no differences between manipulations in the early time point (Control vs. DMS p=0.389; Control vs. DLS p=0.052; Control vs. MC->DLS p=0.522; DMS vs. DLS p=0.213; DMS vs. MC->DLS p=0.759; DLS vs. MC->DLS p=0.104). In the late time point DLS and MC->DLS animals were different from Control and DMS animals (all p<0.001) and from each other (p=0.005). There were no differences between Control and DMS (p=0.3). We note that MC->DLS animals include animals injected with either CAV (n=6) or rAAV (n=3) as the retrograde virus in DLS, and that no significant differences between the two virus groups were detected. See also Fig. S3E for a comparison of the manipulation effects. **E)** Population results for extended training in animals after either DLS lesions or MC->DLS silencing. Shown is the fraction of trials with IPI close to the target (700 ms +/- 20%). **F)** Comparison between DLS injection control animals (replotted from Fig. 2B but split by control type: retrobeads injected in DLS or MC->DLS GFP expression (n=3/3)) and control animals for unspecific, leaky expression of TeLC in motor cortex without Cre expression. Shown is the averaged performance over the course of learning measured as fraction of trials with IPI close to the target (700 ms +/- 20%). A one-way ANOVA revealed no significant differences in the starting performance of the three groups (first 1,000 trials: F(2,7)=2.315; p=0.169; confirmed by Kruskal-Wallis H(2)=4.045; p=0.132). **G)** Trials to reach our learning criterion (see Methods) for the three control groups shown in (F). A one-way ANOVA showed no significant effect of manipulation, (F(2, 7) = 0.03, p=0.97) (confirmed by Kruskal-Wallis test (H(2)=0.118; p=0.943). A pairwise KS-test (with Bonferroni correction) on the cumulative curves also showed no significant differences (p=1 for all comparisons). (Trials to criterion: Control beads: 18375+/-11820; Control MC->DLS GFP: 19198+/-8389; MC TeLC no CRE: 17578+/-5702). *p < 0.05, **p < 0.01, ***p < 0.001.

Supplementary Figure 2

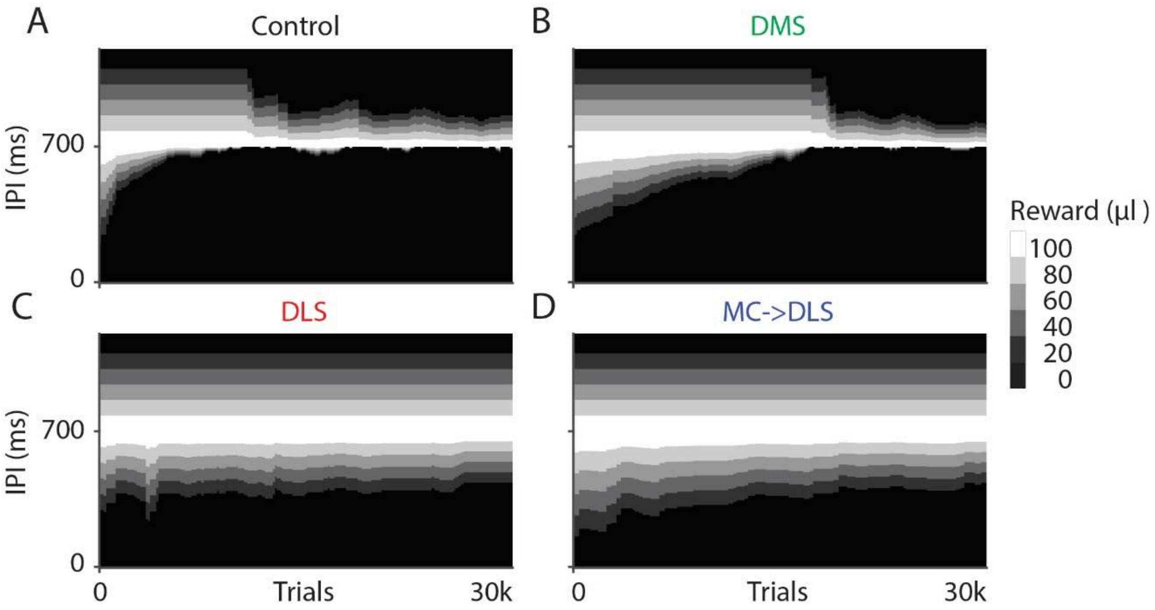

Fig. S2.

Development of the reward landscape for example animals over the course of learning (compare Fig. 1). In order to shape the behavior of experimental animals over the course of training, the reward landscape in our task is automatically adjusted based on the performance of the trained animal. See Methods for a detailed explanation of the training process. **A, B)** As shown in Fig. 1, control animals and DMS-lesioned animals learn our task, which is reflected by the narrowing of the reward landscape over time. **C,D)** DLS-lesioned animals and animals in which motor cortex neurons projecting to the DLS are silenced do not learn our task, which is reflected by the minimal changes in the reward landscape over time.

Supplementary Figure 3

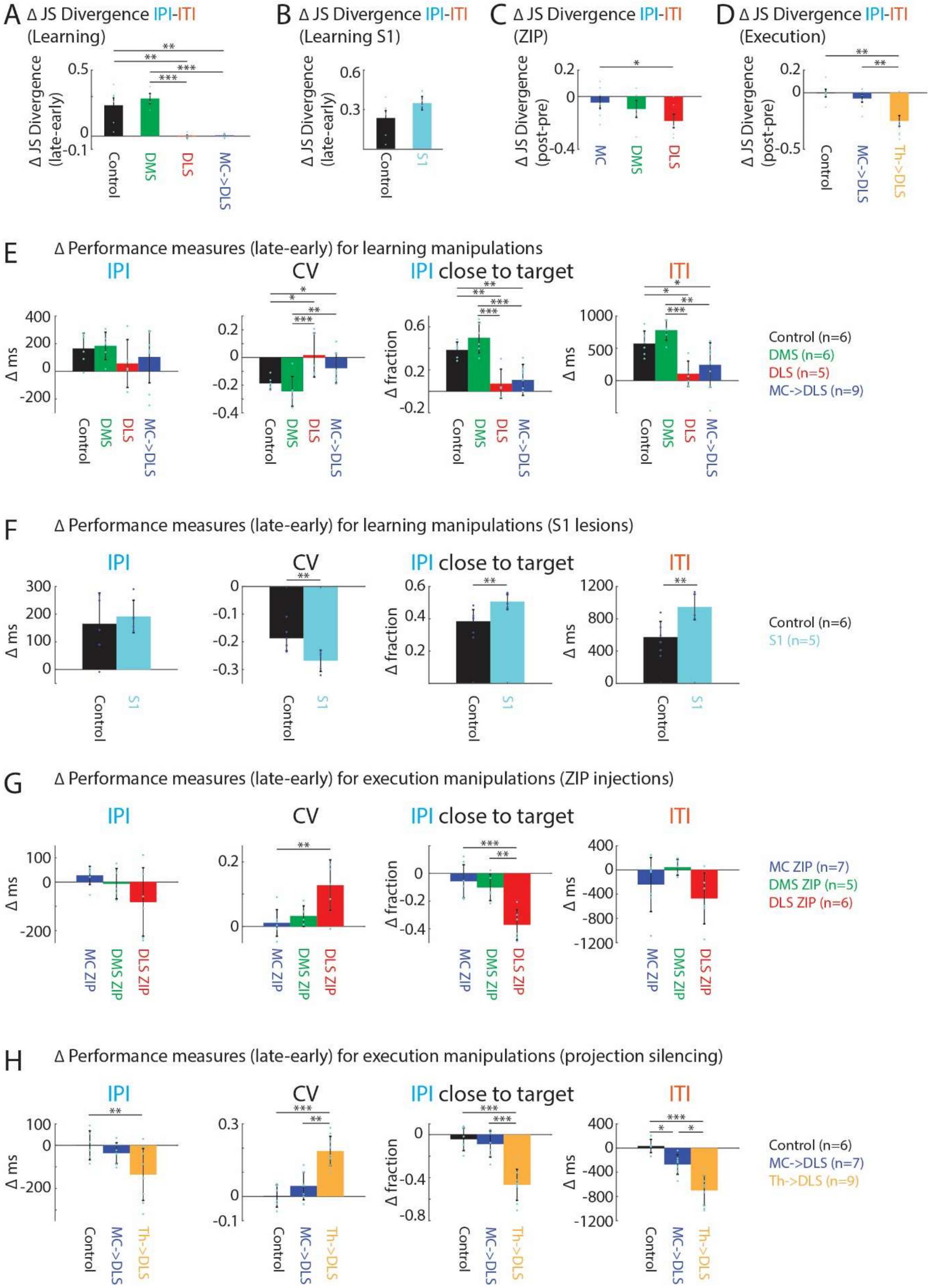

Fig. S3.

Effects of manipulations on performance measures, shown as differences between time points (late-early or post-pre). Comparisons of different performance measures shown throughout the figures are replotted here as differences between early and late or pre and post time points. **A)** Differences in JS Divergence (late-early) for learning manipulations (compare Figure 2E). A Kruskal-Wallis test showed significant differences between the manipulations (H(3)=18.648; p<0.001). Significant differences were found for Control vs. DLS and Control vs. MC-DLS (each p=0.003) as well as for DLS vs. DMS and DMS vs. MC-DLS (each p<0.001). All other comparisons did not show significant differences (Control vs. DMS p=0.312; DLS vs. MC-DLS p=0.335). **B)** Differences in JS Divergence (late-early) for learning manipulations (S1 lesion; compare Figure S4E). A Kruskal-Wallis test showed no significant differences between the manipulations (H(1)=18.648; p=0.1). **C)** Differences in JS Divergence (post-pre) for ZIP manipulations (compare Figure 3D). A Kruskal-Wallis test showed no significant differences between the manipulations (H(2)=4.071; p=0.131). Post-hoc tests found a significant difference between MC and DLS (p=0.02), but no significant differences for all other comparisons (DLS vs. DMS p=0.131; DMS vs. MC p=0.227). **D)** Differences in JS Divergence (post-pre) for projection silencing (compare Figure 4D). A Kruskal-Wallis test showed no significant differences between the manipulations (H(2)=4.071; p=0.131). Post-hoc tests found a significant difference between MC and DLS (p=0.02), but no significant differences for any of the other comparisons (DLS vs. DMS p=0.131; DMS vs. MC p=0.227). **E)** Differences in performance measures (late-early) for learning manipulations (compare Figure S1D). A Kruskal-Wallis test showed no significant differences between the manipulations for the IPI (H(3)=2.032; p=0.566). A Kruskal-Wallis test showed significant differences between the manipulations for the CV (H(3)=13.101; p=0.004). Post-hoc tests found significant differences between Control and DLS (p=0.011), Control and MC->DLS (p=0.044), DLS and DMS (p<0.001) and DMS and MC->DLS (p=0.003). No differences were detected for Control vs. DMS (p=0.183) and DLS vs. MC->DLS (p=0.192). A Kruskal-Wallis test showed significant differences between the manipulations for the IPI close to target (H(3)=17.934; p<0.001). Post-hoc tests found significant differences between Control and DLS (p=0.004), Control and MC->DLS (p=0.005), DLS and DMS (p<0.001) and DMS and MC->DLS (p<0.001). No differences were detected for Control vs. DMS (p=0.261) and DLS vs. MC->DLS (p=0.342). A Kruskal-Wallis test showed significant differences between the manipulations for the ITI (H(3)=15.088; p=0.002). Post-hoc tests found significant differences between Control and DLS (p=0.014), DLS and DMS (p<0.001) and DMS and MC->DLS (p=0.001). No differences were detected for Control vs. DMS (p=0.093), Control vs. MC->DLS (p=0.055) and DLS vs. MC->DLS (p=0.194). **F)** Differences in performance measures (late-early) for learning manipulations (S1 lesion) manipulations (compare Figure S4I). A Kruskal-Wallis test showed no significant differences between the manipulations for the IPI (H(1)=0.133; p=0.715). A Kruskal-Wallis test showed significant differences between the manipulations for the CV (H(1)=6.533; p=0.011). A Post-hoc test found a significant difference between Control and S1 (p=0.005). A Kruskal-Wallis test showed significant differences between the manipulations for the IPI close to target (H(1)=7.5; p=0.006). A Post-hoc test found a significant difference between Control and S1 (p=0.003). A Kruskal-Wallis test showed significant differences between the manipulations for the ITI (H(1)=4.8; p=0.028). A Post-hoc test found a significant difference between Control and S1 (p=0.014). **G)** Differences in performance measures (post-pre) for ZIP manipulations (compare Figure S5D). A Kruskal-Wallis test showed no significant differences between the manipulations for the IPI (H(2)=3.612; p=0.164). A Kruskal-Wallis test showed significant differences between

the manipulations for the CV (H(2)=6.439; p=0.04). Post-hoc tests found significant differences between MC and DLS (p=0.006). No differences were detected for MC vs. DMS (p=0.164) and DLS vs. DMS (p=0.084). A Kruskal-Wallis test showed significant differences between the manipulations for the IPI close to target (H(2)=10.946; p=0.004). Post-hoc tests found significant differences between MC and DLS (p<0.001), DMS and DLS (p=0.007). No difference was detected for MC vs. DMS (p=0.327). A Kruskal-Wallis test showed no significant differences between the manipulations for the ITI (H(2)=4.514; p=0.105). **H)** Differences in performance measures (post-pre) for projection silencing manipulations (compare Figure S6D). A Kruskal-Wallis test showed significant differences between the manipulations for the IPI (H(2)=5.82; p=0.045). Post-hoc tests found significant differences between Control and Th->DLS (p=0.009). No differences were detected for Control vs. MC->DLS (p=0.183) and MC->DLS vs. Th->DLS (p=0.071). A Kruskal-Wallis test showed significant differences between the manipulations for the CV (H(2)=14.82; p<0.001). Post-hoc tests found significant differences between Control and Th->DLS (p<0.001) and between MC->DLS and Th->DLS (p=0.002). No differences were detected for Control vs. MC->DLS (p=0.222). A Kruskal-Wallis test showed significant differences between the manipulations for the IPI close to target (H(2)=14.89; p<0.001). Post-hoc tests found significant differences between Control and Th->DLS (p<0.001) and between MC->DLS and Th->DLS (p<0.001). No differences were detected for Control vs. MC->DLS (p=0.351). A Kruskal-Wallis test showed significant differences between the manipulations for the ITI (H(2)=16.929; p<0.001). Post-hoc tests found significant differences between Control and Th->DLS (p<0.001) and between MC->DLS and Th->DLS (p=0.013) and between Control and MC->DLS (p=0.032). *p < 0.05, **p < 0.01, ***p < 0.001.

Supplementary Figure 4

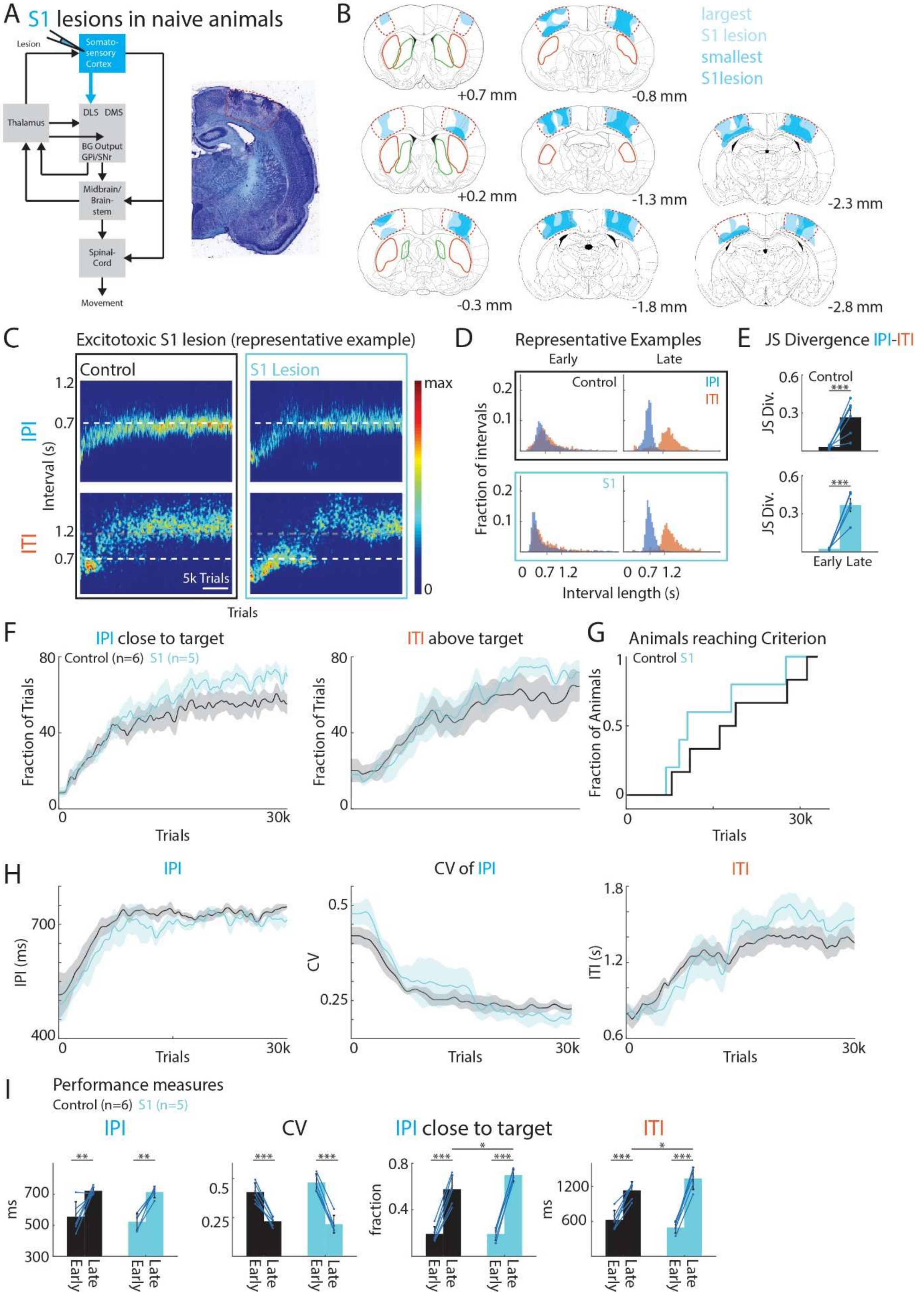

Fig. S4.

Somatosensory cortex is not necessary for learning the skills we train. A) Left: Experimental scheme for excitotoxic lesions of somatosensory cortex (S1). Right: Example lesion of S1. **B)** Extent of S1 lesions (cyan). Light and dark colors indicate the extent of the largest and smallest lesion, respectively. Colored lines indicate the outlines of the DLS and DMS as previously determined by viral labeling of axons from motor cortex and prefrontal cortex, respectively (*16*). Red dotted outlines mark the extent of the targeted area in somatosensory cortex, based on the rat brain atlas (*122*). **C)** Effects of pre-training manipulations on task performance as a function of training (Injection control replotted from Fig. 2, S1 excitotoxic lesion). Shown are heatmaps of IPI and ITI probability distributions for representative animals throughout learning. Colors indicate the probability of the occurrence of a certain interval in a given time-window (Methods). **D)** Distributions of durations between lever-presses for the animals shown in (C) early (first 2000 trials) and late (trials 30,000 to 32,000) in training. **E)** Dissimilarity between the IPI and ITI distributions early and late in training as measured by the JS Divergence. Error bars represent standard error of the mean (SEM). Two-way repeated measures ANOVAs (mixed effects model) were conducted for the JS divergence. Significant effects of time point (early vs. late) ($F(1, 21) = 56.18$, $p<0.001$), but no effect of manipulation, ($F(1, 21) = 1.5$, $p=0.252$) and no significant interaction between manipulation and time point, ($F(1, 21) = 2.12$, $p=0.179$) were found. Simple main effects analysis showed a significant difference between the early and late time points for Control ($p=0.001$) and S1 animals ($p<0.001$). There were no differences between the manipulations in the early (Control vs. S1 $p=0.866$) or late time point (Control vs. S1 $p=0.074$). See also Fig. S3B for further statistical comparison. **F)** Averaged performance across animals for manipulations as in (C). *Left*: Fraction of trials with IPIs close to the target (700 ms +/- 20%). *Right*: Fraction of trials with ITIs above the threshold of 1.2 s. **G)** Fraction of animals reaching the learning criterion (see Methods) over the course of training. Neither an unpaired two-tailed t-test ($p=0.438$), nor a KS-test ($p=0.549$) revealed significant differences between Control and S1 animals. **H)** Population results for performance measures over the course of learning after different manipulations as in (C). IPI: inter-press interval, CV of IPI: Coefficient of Variation of the IPI, ITI: inter-trial interval. **I)** Comparisons of performance measures between different manipulations early and late in training, as in (C). IPI: inter-press interval, CV: Coefficient of Variation of the IPI, IPI close to target: Fraction of trials close to target IPI (700 ms +/- 20%), ITI: inter-trial interval. Early: First 2000 trials in training, Late: trials 30,000 to 32,000 in training. Bars represent means across animals and dots represent means within individual animals. Error bars represent standard error of the mean (SEM). Two-way repeated measures ANOVAs (mixed effects model) were conducted for all measures. For the IPI significant effects of time point (early vs. late) ($F(1, 21) = 41.46$, $p<0.001$), but no effect of manipulation, ($F(1, 21) = 0.81$, $p=0.391$) and no significant interaction between manipulation and time point, ($F(1, 21) = 0.22$, $p=0.648$) were found. Simple main effects analysis showed a significant difference between the early and late time points for Control and S1 animals (both $p=0.001$). There were no differences between the manipulations in the early (Control vs. S1 $p=0.359$) or late time point (Control vs. S1 $p=0.828$). For the CV no significant effects of manipulation, ($F(1, 21) = 0.56$, $p=0.474$), but of time point (early vs. late) ($F(1, 21) = 314.8$, $p<0.001$) and a significant interaction between manipulation and time point, ($F(1, 21) = 9.98$, $p=0.011$) were found. Simple main effects analysis showed a significant difference between the early and late time points both for Control and S1 animals (both $p<0.001$). There were no differences between manipulations in the early (Control vs. S1 $p=0.063$) or late time point (Control vs. S1 $p=0.541$). For the trials with IPI close to target no significant effects of

manipulation, $(F(1, 21) = 1.78, p=0.215)$, but of time point (early vs. late) $(F(1, 21) = 554.65, p<0.001)$ and a significant interaction between manipulation and time point, $(F(1, 21) = 10.32, p=0.01)$ were found. Simple main effects analysis showed a significant difference between the early and late time points both for Control and S1 animals (both $p<0.001$). There were no differences between manipulations in the early (Control vs. S1 $p=0.985$), but in the late time point (Control vs. S1 $p=0.023$). For the ITI no significant effects of manipulation, $(F(1, 21) = 0.21, p=0.654)$, but of time point (early vs. late) $(F(1, 21) = 193.24, p<0.001)$ and a significant interaction between manipulation and time point, $(F(1, 21) = 11.79, p=0.007)$ were found. Simple main effects analysis showed a significant difference between the early and late time points both for Control and S1 animals (both $p<0.001$). There were no differences between manipulations in the early (Control vs. S1 $p=0.171$), but in the late time point (Control vs. S1 $p=0.04$). See also Fig. S3F for further statistical comparison. *$p < 0.05$, **$p < 0.01$, ***$p < 0.001$.

Supplementary Figure 5

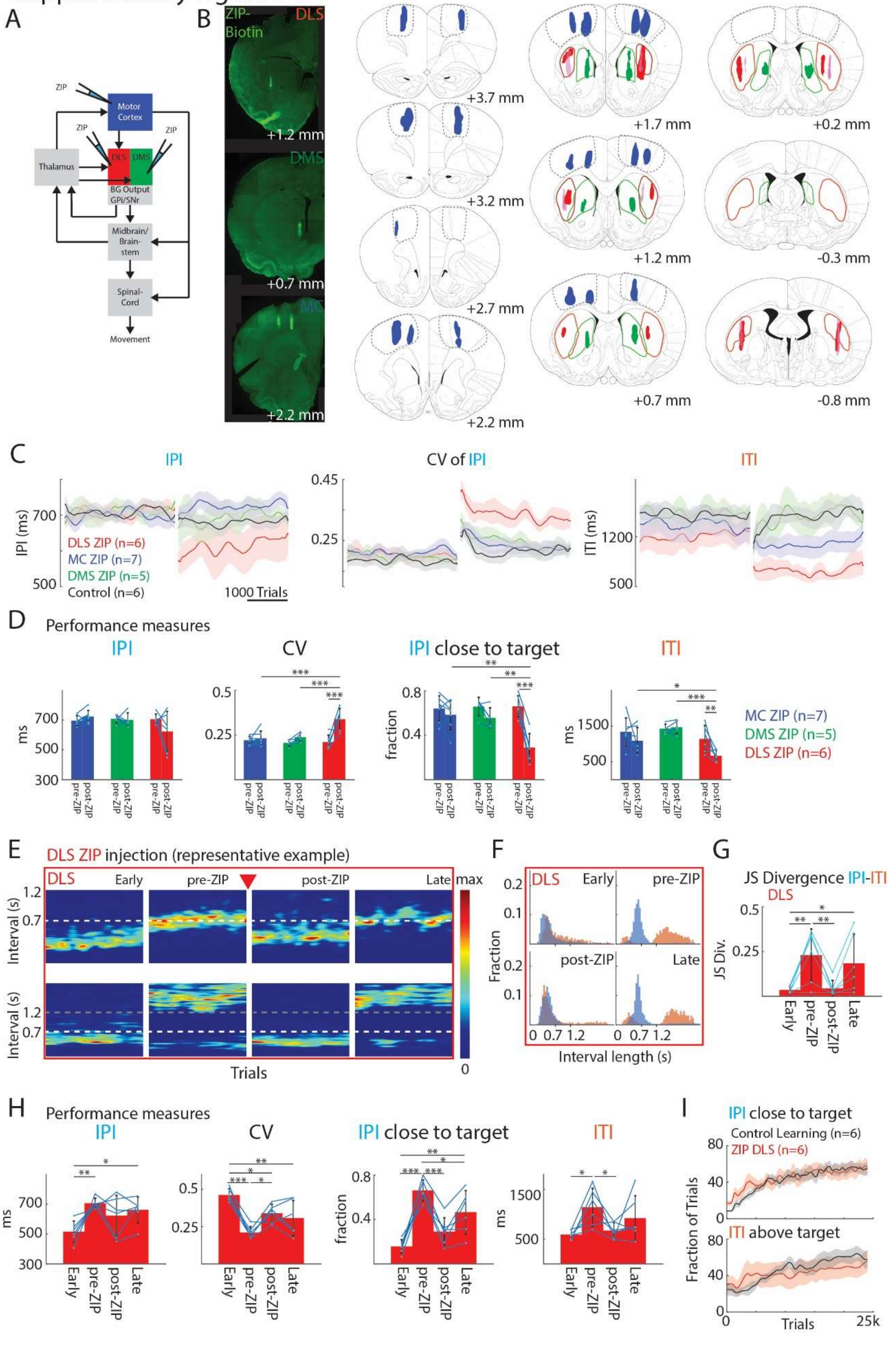

Fig. S5.

Spread of ZIP injections into motor cortex, DLS or DMS and resulting behavioral effects. **A)** Experimental scheme for ZIP injections. **B)** Using biotinylated ZIP and fluorescently labeled avidin, we determined a lower bound for the spread of non-labeled ZIP (with lower molecular weight) in the different target areas. *Left*: Example ZIP-Biotin injections. Bright green indicates spread of ZIP-Biotin labeled with FITC-coupled Avidin (see Methods). The brain tissue is visualized by its auto-fluorescence. *Right*: Extent of ZIP-Biotin injections. Blue: MC, Green: DMS, Red: DLS – dark and light colors indicate injections in different animals. Colored lines indicate the outlines of the DLS and DMS as previously determined by viral labeling of axons from motor cortex and prefrontal cortex, respectively (*16*). Grey outlines mark the extent of the targeted area in motor cortex, based on our previous lesions of motor cortex (*16*, *24*). **C)** Population results for performance measures before and after ZIP injections (as in Fig. 3B; recovery after surgery for 5 days between pre and post). IPI: inter-press interval, CV of IPI: Coefficient of Variation of the IPI, ITI: inter-trial interval. **D)** Comparison of performance measures before and after ZIP injections. IPI: inter-press interval, CV: Coefficient of Variation of the IPI, IPI close to target: Fraction of trials close to target IPI (700 ms +/- 20%), ITI: inter-trial interval. Early: First 2000 trials in training, pre-ZIP: last 2000 trials before ZIP, post-ZIP: first 2000 trials after ZIP, Late: trials 10,000 to 12,000 after ZIP. Bars represent means across animals and dots represent means within individual animals. Error bars represent standard error of the mean (SEM). Two-way repeated measures ANOVAs (mixed effects model) were conducted for all measures. For the IPI no significant effects of manipulation, ($F(2, 15) = 1.74$, $p=0.209$) and of time point (pre vs. post) ($F(1, 15) = 0.9$, $p=0.358$) and no significant interaction between manipulation and time point, ($F(2, 15) = 2.72$, $p=0.121$) were found. For the CV significant effects of manipulation, ($F(2, 15) = 7.27$, $p=0.006$) and of time point (pre vs. post) ($F(1, 15) = 19.11$, $p<0.001$) and a significant interaction between manipulation and time point, ($F(2, 15) = 8.06$, $p=0.004$) were found. Simple main effects analysis showed a significant difference between the pre and post time points for DLS ($p<0.001$), but not for MC ($p=0.607$) and DMS animals ($p=0.211$). There were no differences between manipulations in the pre time point (MC vs. DMS $p=0.516$; MC vs. DLS $p=0.645$; DMS vs. DLS $p=0.836$). In the post time point DLS animals were different from MC and DMS (both $p<0.001$) animals, but there was no difference between MC and DMS animals ($p=0.776$). For trials close to target significant effects of manipulation, ($F(2, 15) = 3.94$, $p=0.042$), and of time point (pre vs. post) ($F(1, 15) = 45.12$, $p<0.001$) and a significant interaction between manipulation and time point, ($F(2, 15) = 14.61$, $p<0.001$) were found. Simple main effects analysis showed a significant difference between the pre and post time points for DLS ($p<0.001$), but not for MC ($p=0.1945$) and DMS animals ($p=0.058$). There were no differences between manipulations in the pre time point (MC vs. DMS $p=0.785$; MC vs. DLS $p=0.744$; DMS vs. DLS $p=0.971$). In the post time point DLS animals were different from MC and DMS (both $p=0.001$) animals, but there was no difference between MC and DMS animals ($p=0.682$). For the ITI significant effects of manipulation, ($F(2, 15) = 7.21$, $p=0.006$) and of time point (pre vs. post) ($F(1, 15) = 6.14$, $p=0.025$), but no significant interaction between manipulation and time point, ($F(2, 15) = 2.53$, $p=0.113$) were found. Simple main effects analysis showed a significant difference between the pre and post time points for DLS ($p=0.008$), but not for MC ($p=0.11$) and DMS animals ($p=0.795$). There were no differences between manipulations in the pre time point (MC vs. DMS $p=0.626$; MC vs. DLS $p=0.253$; DMS vs. DLS $p=0.133$). In the post time point DLS animals were different from MC ($p=0.018$) and DMS ($p<0.001$) animals, but there was no difference between MC and DMS animals ($p=0.057$). See also Fig. S3G for further statistical comparison. **E)** Heatmaps of IPI and

ITI probability distributions of a representative animal before and after DLS ZIP injection (from Fig. 3A) as well as early in training (first 2000 trials) and late after ZIP injection (trials 10,000 to 12,000). **F)** Distributions of durations between lever presses for the animal shown in (E). **G)** JS Divergence as a measure of dissimilarity between the IPI and ITI distributions in the same conditions as in (E). A repeated measures ANOVA revealed significant differences between time points ($F(3,15)=7.86$, $p=0.038$). Post-hoc comparisons (Tukey) showed significant differences between early and pre-ZIP, ($p=0.007$), between pre- and post-ZIP ($p=0.009$) and between early and late ($p=0.04$), but not between all other time points (early vs. post $p=0.998$; pre vs. late $p=0.794$; post vs. late 0.056). **H)** Comparison of performance measures as in (D) for the same conditions in ZIP DLS animals as in (E). Repeated measures ANOVAs were conducted for all performance measures. For the IPI significant differences were found between time points ($F(3,15)=5.64$, $p=0.008$). Post-hoc comparisons (Tukey) showed significant differences between early and pre-ZIP ($p=0.007$) and between early and late ($p=0.039$), but not between all other time points (early vs. post $p=0.158$; pre vs. post $p=0.361$; pre vs. late $p=0.795$; post vs. late $p=0.865$). For the CV significant differences were found between time points ($F(3,15)=13.48$, $p<0.001$). Post-hoc comparisons (Tukey) showed significant differences between all groups (early vs. pre ($p<0.001$), early vs. post ($p=0.034$), early vs. late ($p=0.007$), pre vs. post ($p=0.027$)), except between pre and late ($p=0.117$) and post and late ($p=0.855$). For the trials close to target significant differences were found between time points ($F(3,15)=24.25$, $p<0.001$). Post-hoc comparisons (Tukey) showed significant differences between early and pre-ZIP, pre- and post-ZIP (both $p<0.001$), early and late ($p=0.001$) and pre-ZIP and late ($p=0.031$), but not between early vs. post $p=0.189$, or post vs. late $p=0.061$). For the ITI significant differences were found between time points ($F(3,15)=5.23$, $p=0.011$). Post-hoc comparisons (Tukey) showed significant differences between early and pre-ZIP ($p=0.014$) and pre- and post-ZIP ($p=0.036$), but not between all other time points (early vs. post $p=0.958$; early vs. late $p=0.193$; pre vs. late $p=0.494$; post vs. late $p=0.4$). **I)** Development of the IPI and ITI after ZIP DLS injections, compared to the performance of control animals (replotted from Fig. 2B) over the course of re-learning and initial learning, respectively. *$p < 0.05$, **$p < 0.01$, ***$p < 0.001$.

Supplementary Figure 6

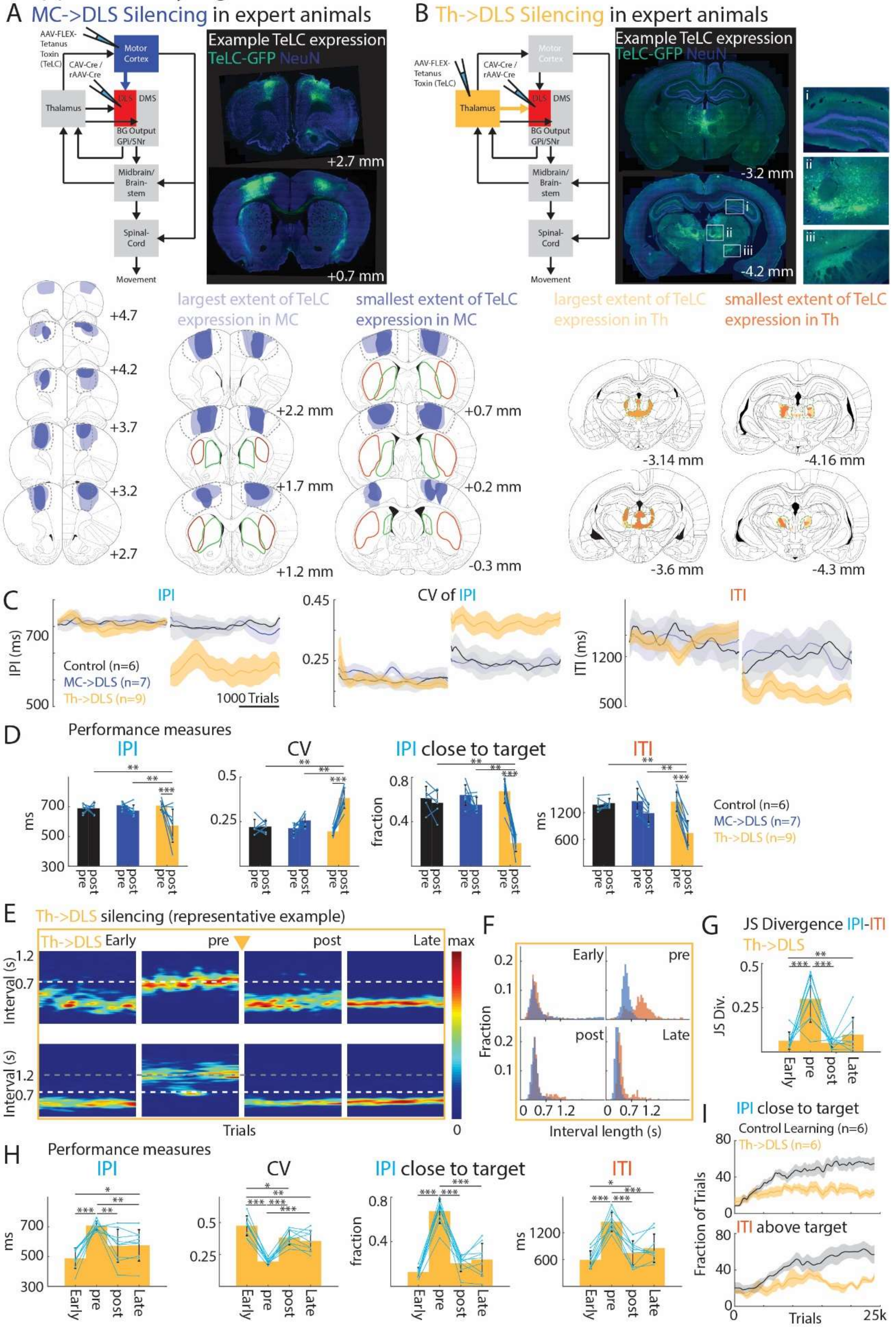

Fig. S6.

Extent of expression of TeLC in motor cortex or thalamus after projection-specific silencing in expert animals and resulting behavioral effects. A) *Top left:* Experimental scheme for projection-specific silencing. *Top right*: Example TeLC-GFP expression in motor cortex. *Bottom*: TeLC-GFP expression extent. Light blue: largest extent of expression of TeLC in motor cortex, Dark blue: smallest extent of expression. Grey outlines mark the extent of the targeted area in motor cortex, based on our previous lesions of motor cortex (*16*, *24*). **B)** *Top left:* Experimental scheme for projection-specific silencing. Our injections target the rostral intralaminar (rILN) nuclei and the perifascicular nucleus (Pf). Note, that this approach also silences the collaterals to other brain areas of the targeted DLS-projecting thalamus neurons. *Top right*: Example TeLC-GFP expression in thalamus. White boxes mark magnified areas shown to the right. *Bottom*: Light yellow: largest extent of expression of TeLC in thalamus, Dark yellow: smallest extent of expression. Green outlines mark the targeted thalamic nuclei (*51*). **C)** Population results for performance measures before and after TeLC expression (as in Fig. 4B; recovery after surgery for 5 days between pre and post). IPI: inter-press interval, CV of IPI: Coefficient of Variation of the IPI, ITI: inter-trial interval. **D)** Comparison of performance measures before and after viral injections. IPI: inter-press interval, CV: Coefficient of Variation of the IPI, IPI close to target: Fraction of trials close to target IPI (700 ms +/- 20%), ITI: inter-trial interval. Early: First 2000 trials in training, pre-silencing: last 2000 trials before silencing, post-silencing: first 2000 trials after silencing, Late: trials 10,000 to 12,000 after silencing. Bars represent means across animals and dots represent means within individual animals. Error bars represent standard error of the mean (SEM). Two-way repeated measures ANOVAs (mixed effects model) were conducted for all measures. For the IPI significant effects of manipulation, ($F(2, 19) = 4.75$, $p=0.021$) and of time point (pre vs. post) ($F(1, 19) = 8.73$, $p=0.008$) and a significant interaction between manipulation and time point, ($F(2, 19) = 4.75$, $p=0.021$) were found. Simple main effects analysis showed a significant difference between the pre and post time points for Th->DLS ($p<0.001$), but not for Control ($p=0.994$) and MC->DLS animals ($p=0.297$). There were no differences between manipulations in the pre time point (Control vs. MC->DLS $p=0.54$; Control vs. Th->DLS $p=0.553$; Th->DLS vs. MC->DLS $p=0.954$). In the post time point, Th->DLS animals were different from Control and MC->DLS (both $p=0.001$) animals; there was no difference between Control and MC->DLS animals ($p=0.619$). For the CV, significant effects of manipulation, ($F(2, 19) = 13.53$, $p<0.001$) and of time point (pre vs. post) ($F(1, 19) = 41.64$, $p<0.001$) and a significant interaction between manipulation and time point, ($F(2, 19) = 23.85$, $p<0.001$) were found. Simple main effects analysis showed a significant difference between the pre and post time points for Th->DLS ($p<0.001$), but not for Control ($p=0.929$) and MC->DLS animals ($p=0.054$). There were no differences between manipulations in the pre time point (Control vs. MC->DLS $p=0.743$; Control vs. Th->DLS $p=0.226$; Th->DLS vs. MC->DLS $p=0.362$). In the post time point Th->DLS animals were different from Control and MC->DLS (both $p=0.001$) animals, but there was no difference between Control and MC->DLS animals ($p=0.125$). For trials close to target significant effects of manipulation, ($F(2, 19) = 12.56$, $p<0.001$) and of time point (pre vs. post) ($F(1, 19) = 51.85$, $p<0.001$) and a significant interaction between manipulation and time point, ($F(2, 19) = 26.15$, $p<0.001$) were found. Simple main effects analysis showed a significant difference between the pre and post time points for Th->DLS ($p<0.001$), but not for Control ($p=0.419$) and MC->DLS animals ($p=0.086$). There were no differences between manipulations in the pre time point (Control vs. MC->DLS $p=0.608$; Control vs. Th->DLS $p=0.222$; Th->DLS vs. MC->DLS $p=0.472$). In the post time point Th->DLS animals were different from Control and MC->DLS (both $p=0.001$)

animals, but there was no difference between Control and MC->DLS animals (p=0.747). For the ITI significant effects of manipulation, (F(2, 19) = 4.79, p=0.021) and of time point (pre vs. post) (F(1, 19) = 56.60, p<0.001) and a significant interaction between manipulation and time point, (F(2, 19) = 27.44, p<0.001) were found. Simple main effects analysis showed a significant difference between the pre and post time points for Th->DLS (p<0.001) and for MC->DLS (p=0.001), but not for Control (p=0.675) animals. There were no differences between manipulations in the pre time point (Control vs. MC->DLS p=0.529; Control vs. Th->DLS p=0.579; Th->DLS vs. MC->DLS p=0.909). In the post time point Th->DLS animals were different from Control and MC->DLS (both p=0.001) animals, but there was no difference between Control and MC->DLS animals (p=0.075). See also Fig. S3H for further statistical comparison. **E)** Heatmaps of IPI and ITI probability distributions of a representative animal before and after thalamostriatal TeLC expression (from Fig. 4A) as well as early in training (first 2000 trials) and late after TeLC expression (trials 10,000 to 12,000). **F)** Distributions of IPIs for the animal shown in (E). **G)** JS Divergence as a measure of dissimilarity between the IPI and ITI distributions in the same conditions as in (E). A repeated measures ANOVA revealed significant differences between time points (F(3,24)=13.37, p=0.006). Post-hoc comparisons (Tukey) showed significant differences between early and pre-silencing, pre- and post-silencing (both p<0.001) and pre-silencing and late (p=0.001), but not between all other time points (early vs. post p=0.993; early vs. late p=0.873; post vs. late p=0.736). **H)** Comparison of performance measures as in (D) for the same conditions in Th->DLS animals as in (E). Repeated measures ANOVAs were conducted for all performance measures. For the IPI significant differences were found between time points (F(3,24)=17.32, p=0.003). Post-hoc comparisons (Tukey) showed significant differences between early and pre-silencing (p<0.001), pre- and post-silencing and pre-silencing and late (both p=0.001) and between early and late (p=0.046), but not between all other time points (early vs. post p=0.054; post vs. late p=0.998). For the CV significant differences were found between time points (F(3,24)=33.19, p<0.001). Post-hoc comparisons (Tukey) showed significant differences between all groups (early vs. pre, pre vs. post, pre vs. late (all p<0.001), early vs. post (p=0.018), early vs. late (p=0.002)), except between post and late (p=0.785). For the trials close to target significant differences were found between time points (F(3,24)=64.03, P<0.001). Post-hoc comparisons (Tukey) showed significant differences between early and pre-silencing, pre- and post-silencing and pre-silencing and late (all p<0.001), but not between all other time points (early vs. post p=0.278; early vs. late p=0.073; post vs. late p=0.884). For the ITI significant differences were found between time points (F(3,24)=36.70, P<0.001). Post-hoc comparisons (Tukey) showed significant differences between early and pre-silencing, pre- and post-silencing, pre-silencing and late (all p<0.001) and early and post (p=0.029), but not between all other time points (early vs. post p=0.311; post vs. late p=0.608). **I)** Development of the IPI and ITI after Th->DLS TeLC expression, compared to the performance of control animals (replotted from Fig. 2B) over the course of re-learning and initial learning, respectively. *p < 0.05, **p < 0.01, ***p < 0.001.

Supplementary Figure 7

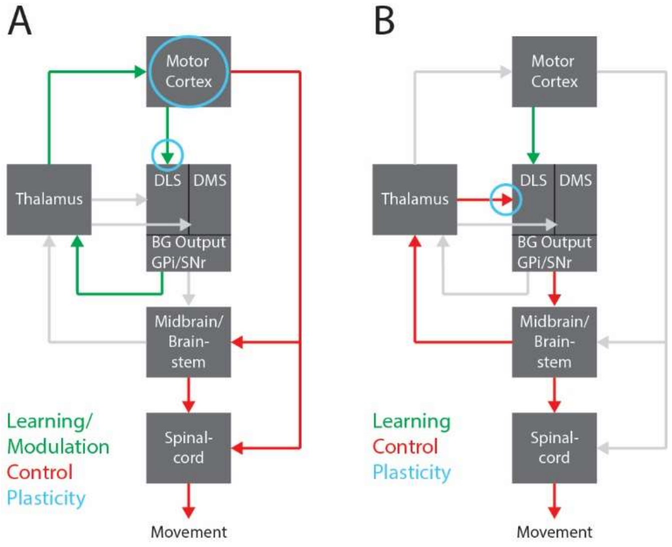

Fig. S7.

Alternative pathways for learning and execution of motor skills. A) Simplified view of the dominant circuit model for the learning and execution of motor skills with motor cortex as a central player. Motor cortex modulates the activity of the Basal Ganglia (BG) via its projection to the dorsolateral striatum (DLS). Over the course of learning this leads to plasticity at corticostriatal synapses. This allows the BG to modulate motor cortex's activity and output via the cortico-BG-thalamo-cortical loop in a more targeted manner. This, in turn, leads to plasticity within motor cortex, allowing it to control the execution of the desired, learned behaviors via its direct projections to motor control centers in the midbrain, brainstem and spinal cord. **B)** Pathway suggested by our results. During learning, motor cortex 'tutors' the BG via its projections to the DLS. At least part of this tutoring may be the gating or induction of synaptic plasticity at thalamic input synapses to the DLS. Once the movement pattern has been learned, the subcortical circuitry, involving the BG-brainstem-thalamo-BG loop, is sufficient to drive movement pattern execution and motor cortex input becomes dispensable. These pathways may interact to different degrees, depending on the behavior and the need for cortical involvement.

Table S1.

IPI close to target

		0-3k	3-6k	6-9k	9-12k	12-15k	15-18k	18-21k	21-24k	24-27k	27-30k
Control vs.	DMS	0.627	0.037	0.013	0.061	0.241	0.356	0.04	0.044	0.02	0.055
	DLS	0.184	0.007	0	0	0	0	0	0	0	0
	MC-DLS	0.362	0.008	0.001	0	0.001	0	0	0	0	0
DMS vs.	DLS	0.386	0.462	0.148	0.011	0.002	0	0	0	0	0
	MC-DLS	0.704	0.72	0.467	0.066	0.026	0	0	0	0	0
DLS vs.	MC-DLS	0.56	0.646	0.377	0.292	0.176	0.242	0.198	0.415	0.439	0.309

ITI above target

		0-3k	3-6k	6-9k	9-12k	12-15k	15-18k	18-21k	21-24k	24-27k	27-30k
Control vs.	DMS	0.993	0.154	0.061	0.087	0.075	0.239	0.51	0.963	0.426	0.556
	DLS	0.723	0.32	0.089	0.046	0.003	0	0	0	0	0
	MC-DLS	0.971	0.359	0.041	0.016	0.001	0	0	0	0	0
DMS vs.	DLS	0.717	0.714	0.926	0.715	0.187	0.007	0.001	0	0	0
	MC-DLS	0.964	0.518	0.99	0.58	0.195	0.003	0.001	0	0	0
DLS vs.	MC-DLS	0.725	0.831	0.929	0.898	0.837	0.906	0.647	0.804	0.716	0.668

IPI

		0-3k	3-6k	6-9k	9-12k	12-15k	15-18k	18-21k	21-24k	24-27k	27-30k
Control vs.	DMS	0.273	0.032	0.015	0.064	0.195	0.63	0.485	0.356	0.494	0.438
	DLS	0.057	0.001	0	0	0	0	0	0	0	0
	MC-DLS	0.557	0.037	0.011	0.037	0.026	0.059	0.064	0.074	0.083	0.137
DMS vs.	DLS	0.387	0.183	0.077	0.015	0.003	0	0	0.002	0.002	0.003
	MC-DLS	0.54	0.788	0.898	0.957	0.418	0.173	0.274	0.435	0.322	0.522
DLS vs.	MC-DLS	0.13	0.09	0.042	0.009	0.013	0.006	0.005	0.008	0.014	0.008

CV

		0-3k	3-6k	6-9k	9-12k	12-15k	15-18k	18-21k	21-24k	24-27k	27-30k
Control vs.	DMS	0.326	0.096	0.036	0.21	0.235	0.982	0.695	0.843	0.613	0.562
	DLS	0.342	0.008	0	0	0	0	0	0	0	0.001
	MC-DLS	0.165	0.002	0.001	0.001	0.002	0.001	0	0.001	0.001	0.002
DMS vs.	DLS	0.989	0.286	0.115	0.005	0.006	0	0	0	0	0
	MC-DLS	0.754	0.201	0.283	0.048	0.062	0.001	0	0.001	0	0
DLS vs.	MC-DLS	0.778	0.961	0.485	0.221	0.227	0.276	0.264	0.167	0.434	0.408

ITI

		0-3k	3-6k	6-9k	9-12k	12-15k	15-18k	18-21k	21-24k	24-27k	27-30k
Control vs.	DMS	0.549	0.059	0.007	0.017	0.035	0.101	0.546	0.934	0.548	0.537
	DLS	0.13	0.015	0.001	0	0	0	0	0	0	0
	MC-DLS	0.507	0.146	0.007	0.003	0.001	0	0	0	0	0
DMS vs.	DLS	0.344	0.516	0.456	0.124	0.017	0.001	0	0	0	0
	MC-DLS	0.995	0.536	0.801	0.709	0.316	0.028	0.003	0	0	0
DLS vs.	MC-DLS	0.307	0.198	0.296	0.187	0.099	0.143	0.097	0.138	0.101	0.092

Table S1: Statistical comparison of learning curves. Repeated measures ANOVAs were used to compare the performance of several animal cohorts (Control, DMS-lesioned, DLS-lesioned, MC->DLS silenced) in different measures over the course of learning. The learning curves on which these comparisons are performed are shown in Figures 2B and S1C. Shown are p values for the individual comparisons in blocks of 3,000 trials. Significant differences between groups ($p<0.05$) are highlighted in gray; a '0' indicates a p value of $p<0.001$.

Table S2.

Figure	Description	Samples	Test	Results
Fig. 2E	JS divergence between IPI and ITI for Control, DMS-lesioned, DLS-lesioned and MC->DLS silenced animals across time points (early, late)	For the 4 groups: 6,6,5,9 rats, respectively	Two-Way repeated measures ANOVA (mixed effect model).<br>Factor 1: Manipulation<br>Factor 2: Time point	Manipulation: $F(3,22)=22.83$, $P<0.001$<br>Time point: $F(1,22)=81.44$, $P<0.001$<br>Interaction: $F(3,22)=28.65$, $P<0.001$
			Simple main effects	P values:<br>Manipulation:<br>Early:<br>1)Cont. vs. DMS: P=0.69<br>2)Cont. vs. DLS: P=0.97<br>3)Cont. vs. MC->DLS: P=0.91<br>4)DMS vs. DLS: P=0.68<br>5)DMS vs. MC->DLS: P=0.58<br>6)DLS vs. MC->DLS: P=0.94<br>Late:<br>1)Cont. vs. DMS: P=0.015<br>2)Cont. vs. DLS: P<0.001<br>3)Cont. vs. MC->DLS: P<0.001<br>4)DMS vs. DLS: P<0.001<br>5)DMS vs. MC->DLS: P<0.001<br>6)DLS vs. MC->DLS: P=0.86<br><br>Time Point:<br>Early vs. Late<br>1)Control: P<0.001<br>2)DMS: P<0.001<br>3)DLS: P=0.92<br>4)MC->DLS: P=0.84

Table S2: Statistical results for Figure 2.

Table S3.

Figure	Description	Samples	Test	Results
Fig. 3D	JS divergence between IPI and ITI for ZIP injections in MC, DMS and DLS across time points (pre, post ZIP)	For the 3 groups: 7,5,6 rats, respectively	Two-Way repeated measures ANOVA (mixed effect model). Factor 1: Manipulation Factor 2: Time point	Manipulation: $F(2,15)=3.46$, $P=0.058$<br>Time point: $F(1,15)=12.99$, $P=0.003$<br>Interaction: $F(2,15)=1.99$, $P=0.17$
			Simple main effects	P values:<br>Manipulation:<br>Pre:<br>1)MC vs. DMS: P=0.07<br>2)MC vs. DLS: P=0.63<br>3)DMS vs. DLS: P=0.17<br><br>Post:<br>1)MC vs. DMS: P=0.26<br>2)MC vs. DLS: P=0.078<br>3)DMS vs. DLS: P=0.009<br><br>Time Point:<br>pre vs. post<br>1)MC: P=0.35<br>2)DMS: P=0.11<br>3)DLS: P=0.003

Table S3: Statistical results for Figure 3.

Table S4.

Figure	Description	Samples	Test	Results
Fig. 4D	JS divergence between IPI and ITI for projection-specific silencing (Control, MC->DLS and Th->DLS) across time points (pre, post silencing)	For the 3 groups: 6,7,9 rats, respectively	Two-Way repeated measures ANOVA (mixed effect model). Factor 1: Manipulation Factor 2: Time point	Manipulation: F(2,19)=8.54, P=0.002<br>Time point: F(1,19)=17.16, P<0.001<br>Interaction: F(2,19)=10.45, P<0.001
			Simple main effects	P values:<br>Manipulation:<br>Pre:<br>1)Cont. vs. MC->DLS: P=0.25<br>2)Cont. vs. Th->DLS: P=0.31<br>3)MC->DLS vs. Th->DLS: P=0.86<br><br>Post:<br>1)Cont. vs. MC->DLS: P=0.053<br>2)Cont. vs. Th->DLS: P<0.001<br>3)MC->DLS vs. Th->DLS: P=0.001<br><br>Time Point:<br>pre vs. post<br>1)Control: P=0.94<br>2)MC->DLS: P=0.25<br>3)Th->DLS: P<0.001

Table S4: Statistical results for Figure 4.

Table S5.

Figure	Description	Samples	Test	Results
Fig. 5D	Comparison of trial-to-trial pairwise correlations within animals after Th->DLS silencing across time points (pre-silencing to pre-silencing, post-silencing to post-silencing, pre-silencing to post-silencing)	4 rats	Repeated measures ANOVA. Factor 1: Time point	Time point: F(2,6)=8.17, P=0.019
			Post-hoc test: Tukey	P values:<br>1)Pre vs. Post: P=0.989 2)Pre vs. Pre-Post: P=0.032<br>3)Post vs. Pre-Post: P=0.027
Fig. 5H	Comparison of trial-to-trial pairwise correlations across animals after Th->DLS silencing across time points (pre-silencing to pre-silencing, post-silencing to post-silencing, pre-silencing to post-silencing)	4 rats	Repeated measures ANOVA. Factor 1: Time point	Time point: F(2,6)=18.4, P=0.003
			Post-hoc test: Tukey	P values:<br>1)Pre vs. Post: P=0.003<br>2)Pre vs. Pre-Post: P=0.673<br>3)Post vs. Pre-Post: P=0.007
Fig. 5J	Comparisons of trial-to-trial pairwise correlations between lever-press trajectories for animals with Th->DLS silencing across time points (early to early, pre to pre, post to post, early to pre, early to post, pre to post)	4 rats	Repeated measures ANOVA. Factor 1: Time point	Time point: F(5,35)=17.13, P<0.001
			Post-hoc test: Tukey	P values:<br>1)Early vs. Pre: P=0.003<br>2)Early vs. Post: P=0.548<br>3)Early vs. Early-Pre: P<0.001<br>4)Early vs. Early-Post: P=0.984<br>5)Early vs. Pre-Post: P=0.003<br>6)Pre vs. Post: P<0.001<br>7)Pre vs. Early-Pre: P=0.545<br>8)Pre vs. Early-Post: P=0.016<br>9)Pre vs. Pre-Post: P=0.995<br>10)Post vs. Early-Pre: P<0.001<br>11)Post vs. Early-Post: P=0.2<br>12)Post vs. Pre-Post: P<0.001<br>13) Early-Pre vs. Early-Post: P<0.001<br>14) Early-Pre vs. Pre-Post: P=0.521<br>15) Early-Post vs. Pre-Post: P=0.018

Table S5: Statistical results for Figure 5.

Movie S1.

Development of idiosyncratic task-specific movement patterns.
Shown are 2 representative trials for 2 different rats trained in our task, early and late in training.
